# Identification of two novel poleroviruses and the occurrence of Tobacco bushy top disease causal agents in natural plants

**DOI:** 10.1038/s41598-021-99320-x

**Published:** 2021-10-26

**Authors:** Song-Tao Tan, Fang Liu, Jing Lv, Qin-Li Liu, Heng-Ming Luo, Yi Xu, Yan Ma, Xiao-Jiao Chen, Ping-Xiu Lan, Hai-Ru Chen, Meng-Ji Cao, Fan Li

**Affiliations:** 1 State Key Laboratory for Conservation and Utilization of Bio-Resources in Yunnan, Yunnan Agricultural University, Kunming, 650201 China; 2 Department of Plant Pathology, Nanjing Agricultural University, Nanjing, 210095 China; 3 National Citrus Engineering Research Center, Citrus Research Institute, Southwest University, Chongqing, 400712 China

**Keywords:** Microbiology, Plant sciences

## Abstract

Tobacco bushy top disease (TBTD) is a devastating tobacco disease in the southwestern region of China. TBTD in the Yunnan Province is often caused by co-infections of several plant viruses: tobacco bushy top virus (TBTV), tobacco vein distorting virus (TVDV), tobacco bushy top virus satellite RNA (TBTVsatRNA) and tobacco vein distorting virus-associated RNA (TVDVaRNA). Through this study, two new poleroviruses were identified in two TBTD symptomatic tobacco plants and these two novel viruses are tentatively named as tobacco polerovirus 1 (TPV1) and tobacco polerovirus 2 (TPV2), respectively. Analyses of 244 tobacco samples collected from tobacco fields in the Yunnan Province through RT-PCR showed that a total of 80 samples were infected with TPV1 and/or TPV2, and the infection rates of TPV1 and TPV2 were 8.61% and 29.51%, respectively. Thirty-three TPV1 and/or TPV2-infected tobacco samples were selected for further test for TBTV, TVDV, TBTVsatRNA and TVDVaRNA infections. The results showed that many TPV1 and/or TPV2-infected plants were also infected with two or more other assayed viruses. In this study, we also surveyed TBTV, TVDV, TBTVsatRNA and TVDVaRNA infections in a total of 1713 leaf samples collected from field plants belonging to 29 plant species in 13 plant families and from 11 provinces/autonomous regions in China. TVDV had the highest infection rates of 37.5%, while TVDVaRNA, TBTV and TBTVsatRNA were found to be at 23.0%, 12.4% and 8.1%, respectively. In addition, TVDV, TBTV, TBTVsatRNA and TVDVaRNA were firstly detected of co-infection on 10 plants such as broad bean, pea, oilseed rape, pumpkin, tomato, crofton weed etc., and 1 to 4 of the TBTD causal agents were present in the samples collected from Guizhou, Hainan, Henan, Liaoning, Inner mongolia and Tibet autonomous regions. The results indicated that TBTD causal agents are expanding its host range and posing a risk to other crop in the field.

## Introduction

Tobacco bushy top disease (TBTD) is one of the important tobacco diseases and is caused by multiple causal agents. TBTD was first reported in Zimbabwe, based on its disease symptoms and vector transmission way^1^. It was reported that the TBTD in China was caused by tobacco bushy top virus (TBTV)^2^, tobacco vein distorting virus (TVDV)^3^, tobacco bushy top virus satellite RNA (TBTVsatRNA)^4^ and tobacco vein distorting virus associated RNA (TVDVaRNA)^5^. The occurrence of TBTD in Yunnan Province, China, was first recorded in 1993^6^. Later, TBTD was found in multiple tobacco growing regions such as Chuxiong, Baoshan and Dali in Yunnan Province^7^. The overall incidences of naturally infected tobaco plants were 91%, 32.5%, 35.7%, 100%, 42.5% and 100% in 1993 to 1998, respectively. The total infected crop was approximately 51,300 ha and harvest losses were estimated to exceed US$ 33 million till 2001^7^. The devastating disease has become a limiting factor in the production of tobacco in the province.

In 2002, TBTV and TVDV were found in diseased tobacco plants and aphid vectors through RT-PCR using degenerate primers for umbraviruses or poleroviruses^8^. In other two studies, TBTVsatRNA and TVDVaRNA were identified in tobacco plants with TBTD symptoms in China through double stranded RNA (dsRNA) analysis and sequencing^4,5^. *Tobacco bushy top virus* is a member in the genus *Umbravirus*, family *Tombusviridae* and *Tobacco vein distorting virus* is a member in the genus *Polerovirus*, family Solemoviridae^9^. In a recent study, TBTD was found in several tobacco species in Ethiopia and these diseased plants were infected with *Ethiopian tobacco bushy top virus*, a new member of genus *Umbravirus*, *Potato leafroll virus*, a member in the genus *Polerovirus*, family Solemoviridae, and an Ethiopian satellite RNA (ETBTVsatRNA)^10^. To date, how TBTV, TVDV, TVDVaRNA and TBTVsatRNA co-infect tobacco and other natural plants in fields and whether TBTD plants have other unidentified viruses are remain unknown.

Umbraviruses (members in genus *Umbravirus*) are a group of positive-sense single-stranded RNA viruses. Umbravirus RNA does not have a 5′ cap and a 3′ poly(A) tail, and does not encode a capsid protein (CP)^9^, and thus doesn’t form typical virion structure. For successful aphid transmission, umbraviruses need the presence of their helper viruses, mostly a polerovirus or a enamovirus. For instance, aphid transmission of groundnut rosette virus (GRV) depends on the presence of groundnut rosette assistor virus (GRAV, polerovirus) and groundnut rosette virus satellite RNA (GRVsatRNA)^11^. Aphid transmission of pea enation mosaic virus 2 (PEMV-2) requires the presence of pea enation mosaic virus 1 (PEMV-1, enamovirus)^12,13^.

Poleroviruses (members in genus *Polerovirus*) are also positive-sense single-stranded RNA viruses. The genome size of poleroviruses range from 5.6 to 6.0 kilobases (kb). Polerovirus RNA does not have a Poly(A) tail and a tRNA-like structure at its 3′ end. *Polerovirus*, *Polemovirus, Sobemovirus* and *Enamovirus* are the four genera in the family Solemoviridae, there were 5 (*Enamovirus*), 20 (*Sobemovirus*), 1 (*Polemovirus*), 26 (*Polerovirus*) species in these four genera. Polerovirus genomic RNA contains 7 ORFs. Three ORFs (e.g., ORF0, ORF1 and ORF1-ORF2) are translated from the 5′ half of the genomic RNA while ORF3a, ORF3, ORF4 and ORF3-ORF5 are expressed from the subgenomic RNA transcribed from the 3′ half of the genomic RNA. Most poleroviruses have limited natural host range, mainly in family Solanaceae, and some in family Amaranthaceae and family Cruciferae^14^. While some poleroviruses have a broad host range, for example, beet western yellows virus (BWYV) infects more than 150 plant species in over 20 families^9^.

A recent study showed that many plants without typical TBTD symptoms were also infected with one to three TBTD causal agents, while the tobacco plants infected with all the four causal agents did^15^, indicating that the development of TBTD symptoms in plants is a complicated process and is associated with all the four reported viruses. Earlier studies on the TBTD in China were focused mainly on tobacco host plant and in Yunnan Province. In recent years, one or more TBTD causal agents have been identified in other plant species and in other regions of China^16–18^. To further investigate the TBTD causal agents in China, we performed High throughput sequencing from two tobacco plants showing typical TBTD symptoms. Result of this analysis showed that, in addition to the four known agents, these two plants were also infected two new poleroviruses: tobacco polerovirus 1 (TPV1) and tobacco polerovirus 2 (TPV2). We then tested 1713 samples from 29 plant species in 11 provinces/autonomous regions in China by RT-PCR using primers specific for TBTV, TVDV, TVDVaRNA or TBTVsatRNA. The results presented in this paper should allow us to better understand TBTD, and the potential risk of TBTD outbreak in many crop species. These knowledge should benefit the development of an effective management strategy for this diseases.

## Materials and methods

### Field samples collection

Two tobacco plants (referred to as YBSh and YKMPL) showing TBTD symptoms were collected from two different tobacco planting fields in Baoshan and Kunming Cities, Yunnan Province, China in 2015 and 2016 respectively, and were stored in insect-proof greenhouse. To survey the occurrence of TBTV, TVDV, TBTVsatRNA and TVDVaRNA in fields, 1550 virus-like leaf samples were collected from plants belonging to 29 species in 13 families in Yunnan Province from 2013 to 2018. And 65 pepper plants with virus-like symptoms were collected from Guizhou, Liaoning, Henan, Hainan, Shandong, Zhejiang, Hubei Province and Tibet, Inner Mongolia Autonomous Region, China, and 83 tomato plants with virus-like symptoms were collected from Liaoning, Henan, Hainan, Shandong, Shaanxi, Zhejiang Province, and Tibet, Inner Mongolia Autonomous Region. In addition, 11 crofton weed leaf samples were also collected from Guizhou, 1 purple perilla and 3 dahlia were collected from Liaoning (Supplementary Table 1). The voucher IDs form of plant that are not cultivated on commercial scale was shown in Table 1.

**Table 1:** Sample ID of Crofton weed, Purple Perilla & Dahlia.

Plant species	Sample ID
Crofton weed	YNZJZL-1
	YNZJZL-2
	YNZJZL-3
	YNZJZL-4
	YNZJZL-5
	YNZJZL-6
	GZZJZL-1
	GZZJZL-2
	GZZJZL-3
	GZZJZL-4
	GZZJZL-5
	GZZJZL-6
	GZZJZL-7
	GZZJZL-8
	GZZJZL-9
	GZZJZL-10
	GZZJZL-11
Purple Perilla	LNZS-1
Dahlia	LNDL-1
	LNDL-2
	LNDL-3

### High throughput sequencing and data analyses

To verify the viruses infecting the two TBTD-symptoms tobacco field samples, the YBSh and YKMPL leaf samples were collected and then quick-frozen by liquid nitrogen and stored at − 80 °C tentatively. The two samples were sent to Biomarker Technologies (Beijing, China) for High throughput sequencing (HTS) RNA-Seq sequencing after depletion of the rRNAs with Epicentre Ribo-ZeroTM kit, which was then sequenced using the Illumina HiSeq X-ten platform with PE150 bp (Illumina, San Diego, CA, USA). Sequence data were analyzed using CLC Genomic Workbench 9.5 (QIAGEN, Hilden, Germany) as described^19^. Reads without sequence similarity and not mapping to the reference tobacco genome were assembled de novo by Trinity program. The generated contigs were used as queries for BLAST searches; contigs that were not identified as sequences already included in the databases were sorted out as candidate genomic fragments of the novel virus.

### Full genome amplification and sequencing of the viruses in samples YBSh and YKMPL

The sequence gaps between the aligned contigs were filled by RT-PCR using virus-specific primers. The 5′- and 3′-end sequences of TBTV, TVDV, TBTVsatRNA and TVDVaRNA were determined by the rapid amplification of cDNA ends (RACE) technique using SMARTer RACE 5′/3′ Kit (Clontech, USA).

The genomic sequences of the viruses were assembled using the DNASTAR 7.0 package (DNASTAR Inc., Madison, WI, USA), and then submitted to the GenBank database in NCBI. To characterize the two newly identified viruses, ORF finder software (https://www.ncbi.nlm.nih.gov/orffinder/) was used to predict their ORFs. Pairwise comparisons were performed using the EMBOSS Needle Pairwise Sequence Alignment software available at the http://www.ebi.ac.uk/Tools/psa/emboss_needle/nucleotide.html. Phylogenetic relationship between the two new viruses and the other known poleroviruses was determined by the MEGA 5.0 software. The sequences were all linearized at the start of the RdRp gene and then aligned using MEGA 5.0. The alignments were used to infer Neighbor joining trees in MEGA 5.0 with P-distance model and 1000 bootstrap replicates as described^20^.

### Viruses detection and sequence confirmation of the two new poleroviruses in the field tobacco samples

To detect the two newly identified poleroviruses in the field-collected samples, total RNAs were extracted from 817 tobacco leaf samples using the TRIpure Reagent (Bioteke, Beijing, China) for reverse transcription-polymerase chain reaction (RT-PCR). RT-PCR reactions were performed using specific primers based on the two new poleroviruses sequences (Supplementary Table 2) and PrimeScript™ One-Step RT-PCR Kit Ver. 2 (TaKaRa Biotechnology, Dalian, China) as instructed. Positive RT-PCR products were gel purified and cloned individually into the pMD19-T vector (TaKaRa). The resulting plasmid DNAs were sequenced by BGI (BGI, Guangzhou, China) and the resulting viral sequences were assembled using the DNASTAR 7.0 package (DNASTAR Inc., Madison, WI, USA).

### Detection of TBTV, TVDV, TBTVsatRNA and TVDVaRNA infections in the field-collected samples

To determine the occurrence of TBTV, TVDV, TBTVsatRNA and TVDVaRNA in the field samples, virus specific primers (Supplementary Table 2) were used and the four viruses were simultaneously detected in the field-collected samples through multiplex RT-PCR as previously described^15^.

### Identification and preservation of plant samples

The species identification was carried out with the help of Dr. Yunheng Ji, (Kunming Institute of Botany, Chinese Academy of Sciences). All samples were stored in the Virus Laboratory (State Key Laboratory for Conservation and Utilization of Bio-Resources in Yunnan, Yunnan Agricultural University).

### Ethical guidelines

All the protocols involving plant adhered to relevant ethical guidelines.

## Results

### Symptomology of TBTD and viruses detected in the two TBTD affected tobacco plants by HTS

**Figure 1:**
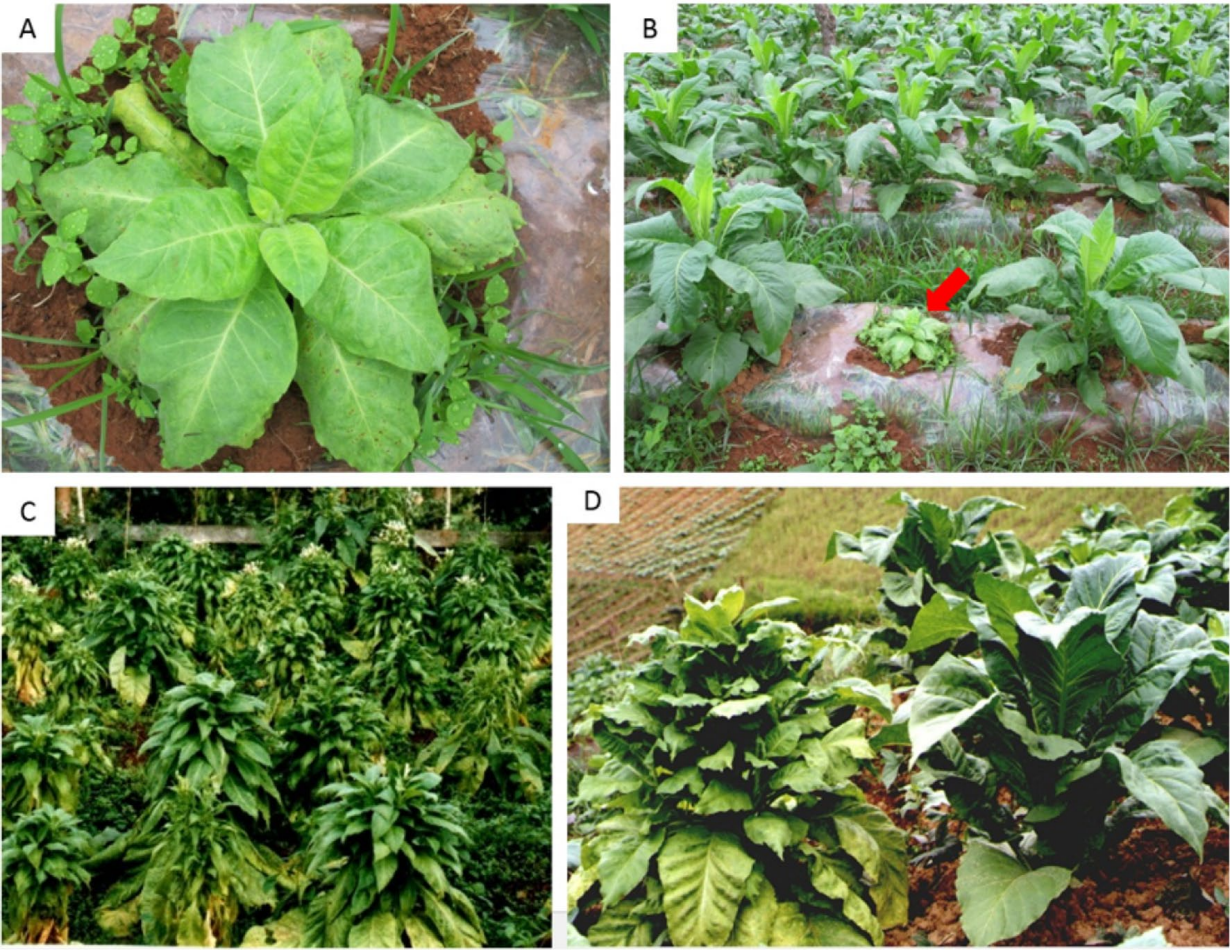
Symptoms of TBTD-affected flue-cured tobacco cultivar K326 in field. Symptoms of an early infection on tobacco plant including chlorosis, internode shortening and serious stunting (**A**). Early infected plant (indicated by the red arrow) and healthy looking tobacco plants (**B**). Late infection symptoms on tobacco plants showing lateral branches proliferation, small leaves, foliar yellowing or chlorosis, stunting (**C**). Late infected plant (left) and healthy looking tobacco plant (right) (**D**).

The most frequently observed TBTD symptoms on flue-cured tobacco (*Nicotiana tobacum*) in field include small leaves, irregular necrotic lesions on leaves, yellowing or chlorosis, internode shortening and stunting (Fig. 1). Diseased tobacco plants became chlorosis, significantly stunted and failed to flower when infected at an early stage (Fig. 1A, B), the fully infected plants were thus unmarketable. While late infections developed lateral branches proliferation, small leaves, foliar yellowing or chlorosis, stunting and without impaired flowering (Fig. 1C, D), only the lower and uninfected leaves were marketable from these plants.

Two TBTD symptoms tobacco plants YBSh and YKMPL were collected for HTS RNA-Seq sequencing to further verify the viruses infecting the TBTD-symptoms field tobacco plants. A total of 36,744,321 and 33,356,805 clean RNA reads were obtained from the YBSh and YKMPL samples through HTS after removing the failed reads, respectively. These clean reads were assembled using the Illumina HiSeq X-ten platform with PE150 bp and the CLC Genomic Workbench 9.5 (QIAGEN, Beijing, China) as described^19^. In which 56,964 contigs for YBSh and 52,916 contigs for YKMPL larger than 200 bp were assembly generated by de novo*.* A total of 122,028 reads were associated with TBTVsatRNA, 22,370 reads were associated with TVDVaRNA, 1775 reads were associated with TBTV, 15,430 reads were associated with TVDV, 6397 reads were associated with TPV1 and 8594 reads were associated with TPV2 in sample YBSh. A total of 116,779 reads were related with TBTVsatRNA, 1124 reads were related with TVDVaRNA, 14,388 reads were related with TBTV, 2923 reads were related with TVDV, 277 reads were related with TPV1 and 339 reads were related with TPV2 in sample YKMPL. The resulting contigs were subjected to BlastX and BlastN searches against the databases at the NCBI, results revealed that both tobacco plants YBSh and YKMPL infected with six viruses.

The results showed that both the YBSh and YKMPL tobacco samples were co-infected with 6 different viral agents of TBTV, TBTVsatRNA, TVDV, TVDVaRNA and two novel poleroviruses (designated as isolates YBSh and YKMPL, respectively). Based on the results of sequence alignment, we tentatively named these two new poleroviruses as tobacco polerovirus 1 (TPV1) and tobacco polerovirus 2 (TPV2). To validate the reliability of HTS results, the total RNA samples isolated from the YBSh, YKMPL samples as well as healthy tobacco sample were analyzed by RT-PCR. TPV1 and TPV2 detection primers were designed according to the virus contigs identified through HTS, while the primers and multiplex one-step RT-PCR used to detect TBTV, TBTVsatRNA, TVDV, TVDVaRNA were described previously^15^. The results showed that the PCR products representing the six viruses were indeed present in the YBSh and YKMPL samples, but not in the sample from healthy plant (Figure S1A–C).

### Characterization of the TBTV, TVDV, TVDVaRNA and TBTVsatRNA of YBSh and YKMPL isolates

To obtain the full-length genome sequences of TBTV, TVDV, TVDVaRNA and TBTVsatRNA of YBSh and YKMPL isolates, overlapping amplicons cloning strategy was used in a series of sequential RT-PCR with virus specific primers designed according to the virus sequences from HTS. The primers, amplification strategies (position in the virus genomes), size of the amplicons, and specify chemistry for sequencing were add to the supplementary materials (Fig. S2; Table S3). At least three clones from each amplicon were sequenced on both strands using M13 forward and reverse primers as well as specific sequencing primers if necessary. Results showed that the full-length genome sequences of the two TBTV isolates both were determined to be 4152 nucleotides (GenBank accession number: TBTV-YBSh, MW579556; TBTV-YKMPL, MW579557). Pairwise comparison of the complete nucleotide sequences of different TBTV isolates showed that TBTV-YBSh shared 97.0% nt sequence identity with TBTV-YKMPL. TBTV-YBSh and TBTV-YKMPL shared 94.7% (TBTV-MD-II, KM067277) to 98.7% (TBTV-MD-I, KM016225) and 85.6% (TBTV-YDHo, KX216406) to 98.8% (TBTV-MD-I) nt sequence identity with other TBTV isolates available in GenBank. The full-length genomic sequences of the two TVDV isolates were determined to be 5920 nt (GenBank accession number: TVDV-YBSh, MW579560; TVDV-YKMPL, MW579561). Pairwise comparison of the complete nucleotide sequences of different TVDV isolates showed that TVDV-YBSh shared 99.5% nt sequence identity with TVDV-YKMPL. TVDV-BSh and TVDV-KMPL shared 97.6% and 97.4% nt sequence identity with TVDV (EF529624). The full-length TVDVaRNA-YBSh and TVDVaRNA-YKMPL sequences were detemined to be 2971 nts (GenBank accession number: TVDVaRNA-YBSh, MW579562; TVDVaRNA-YKMPL, MW579563). Pairwise comparison of the complete nucleotide sequences of different TVDVaRNA isolates showed that TVDVaRNA-YBSh shared 98.3% nt sequence identity with TVDVaRNA-YKMPL. TVDVaRNA-YBSh and TVDVaRNA-YKMPL share 95.8% and 94.2% nt sequence identity with TVDVaRNA (EF529625). The full-length TBTVsatRNA-YBSh and TBTVsatRNA-YKMPL sequences has determined to be 824 nts (GenBank accession number: TBTVsatRNA-YBSh, MW579558; TBTVsatRNA-YKMPL, MW579559). Pairwise comparison of the complete nucleotide sequences of different TBTVsatRNA isolates showed that TBTVsatRNA-YBSh shared 90.8% nt sequence identity with TBTVsatRNA-YKMPL. TBTVsatRNA-YBSh and TBTVsatRNA-YKMPL shared 98.5% (TBTVsatRNA Longling, KU997687) and 89.0% (TBTVsatRNA YN4, AM238656) nt sequence identity with other TBTVsatRNA isolates available in GenBank. These results indicate that these four viruses are identical to the TBTD causal agents reported previously.

### Sequence analysis and genome organization of TPV1 and TPV2

**Figure 2:**
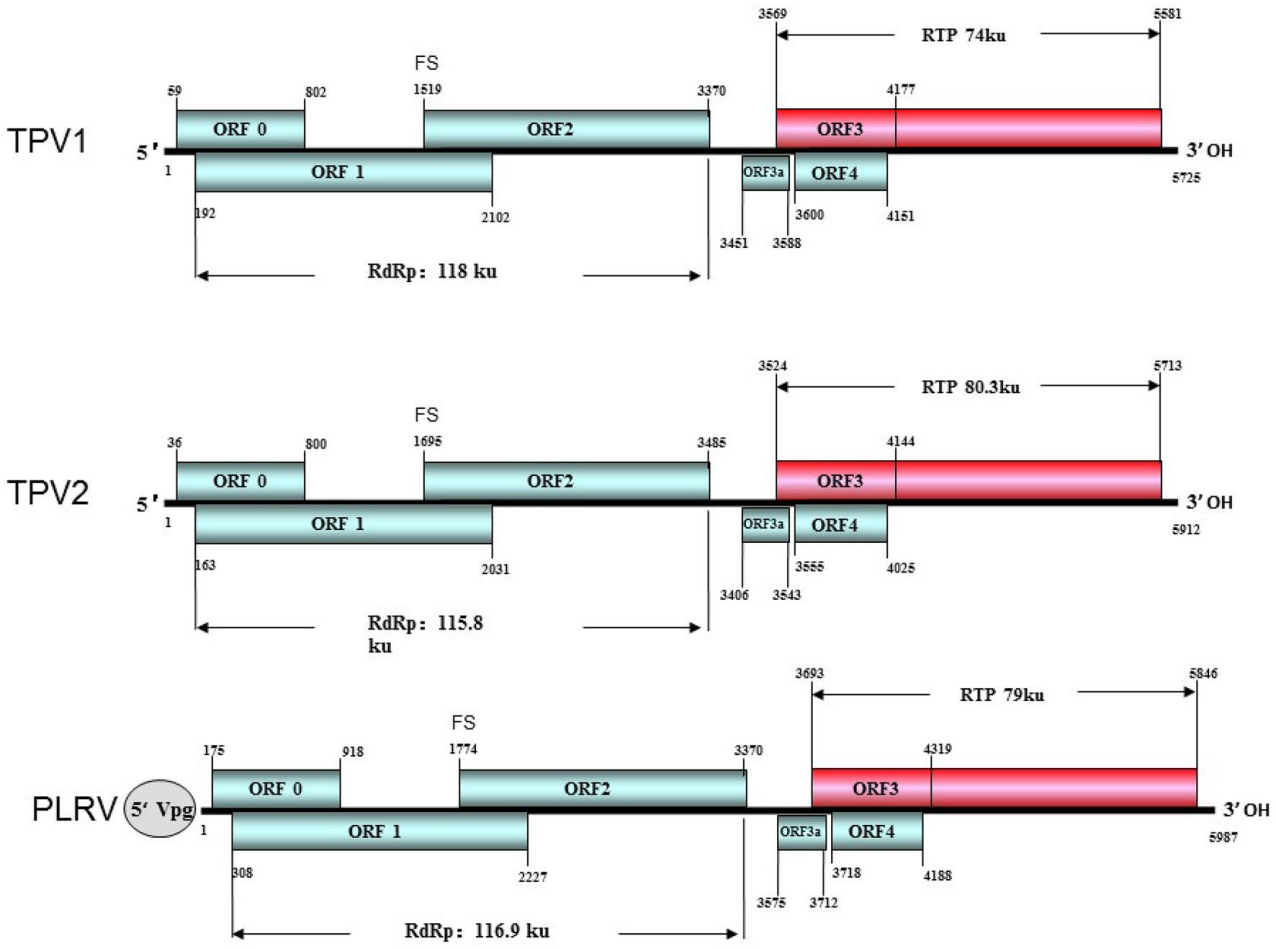
Genome structures of TPV1, TPV2 and PLRV.

Two new poleroviruses, TPV1 and TPV2, were found both in the YBSh and YKMPL field samples through HTS. The nearly full-length genome sequences of isolates TPV1-YBSh (GenBank accession number: MW579552), TPV1-YKMPL (GenBank accession number: MW579553) and TPV2-YBSh (GenBank accession number: MW579554), TPV2-YKMPL, (GenBank accession number: MW579555) were confirmed to be 5722nt, 5725nt and 5907nt, 5912nt, respectively, by series of sequential RT-PCR and SMARTer®RACE 5′/3′ kit (Clontech Laboratories. lnc, USA) with virus specific primers based on the HTS data followed by Sanger sequencing. Pairwise comparison results of the nearly complete sequences showed that TPV1-YBSh shared 99.7% nt identity with TPV1-YKMPL, and TPV2-YBSh shared 98.9% nt identity with TPV2-YKMPL, respectively. The genomic sequences of TPV1-YBSh and TPV2-YBSh, therefore, were used in the subsequent sequence analysis. The genomic nucleotide sequence identity between TPV1 and TPV2 is 54.2%, suggesting they are two distinct species. Blast search results indicated that TPV1 and TPV2 had the highest nt sequence identity with the known poleroviruses. The genome structures of TPV1 and TPV2 were predicted using the ORFfinder software (https://www.ncbi.nlm.nih.gov/orffinder). The genomic organization and structure of TPV1 and TPV2 is typical of poleroviruses when comparing with PLRV, and both TPV1 and TPV2 contain seven ORFs: ORF0, ORF1, ORF1-ORF2, ORF3a, ORF3, ORF4 and ORF3-ORF5 (Fig. 2). The ORF0 of TPV1 has 744 nts and encodes a 28 kDa P0 protein. The ORF1 has 1911 nts and encodes a 69.4 kDa P1 protein, and the ORF1-ORF2 frame has 3179 nts and encodes a 118 kDa P1–P2 protein through an -1 frameshift translation strategy. The intergenic region (IR) between ORF2 and ORF3a is 81 nts. The ORF3a has an ATA initiation codon and its 3′-terminal 40 nts overlaps with the 5′-termial of ORF3. The ORF3a encodes a 5.1 kDa P3a protein. The ORF3 has 609 nts and encodes a 22.4 kDa P3 protein. The ORF4 has 552 nts, overlapping completely with ORF3, and encodes a 17 kDa P4 protein. The ORF5 has no initiation codon for independent translation. The ORF3 and ORF5 are predicted to form an ORF3-ORF5 frame encoding a 74 kDa read-through protein (RTP)  through a read-through translation strategy. The 3′ end untranslated region (UTR) of TPV1 has 145 nts. Analysis of the nearly full-length genome sequence of TPV2 showed that in contrast to TPV1, TPV2 ORF0 has 765 nts and encodes a 28 kDa P0 protein. The ORF1 contains 1869 nts and encodes a 68.5 kDa P1 protein and the ORF1-ORF2 frame has 3323 nts and encodes a 115.8 kDa P1–P2 frameshift translated protein. The IR reigon between the ORF2 and ORF3a has 83 nts. The 3′-terminal 20 nts of ORF3a overlaps with the 5′-terminal of ORF3. The ORF3a contains 138 nts and encodes a 5.1 kDa P3a protein. The ORF3 contains 621 nts and encodes a 22.8 kDa P3 protein. The ORF4 contains 471 nts and encodes a 20.4 kDa P4 protein. The ORF3-ORF5 frame encodes an 80.3 kDa read-through protein, and the 3′-terminal UTR of TPV2 has 200 nts (Fig. 2).

### Sequence comparison of TPV1 and TPV2 with other poleroviruses

To further characterize TPV1 and TPV2, the nearly full-length sequences of TPV1 and TPV2 were compared with the corresponding region of the poleroviruses available in GenBank. The results showed that TPV1 shared the nt sequence identity from 50.4% (sugarcane yellow leaf virus, ScYLV, AF157029) to 79.1% (tobacco virus 2, TV2, KY038943) among the 19 poleroviruses (Table 2). The nucleotide and amino acid sequences of different ORFs were compared for TPV1 with 24 selected poleroviruses, the results revealed that TPV1 shared nt sequence identity of 49.2% (TPV2) to 97.3% (TV2, KY038943) in ORF0 with other poleroviruses, 29.6% (maize yellow dwarf virus-RMV, MYDV-RMV, KC921392) to 96.4% (TV2) in ORF1, 57.4% (suakwa aphid-borne yellows virus, SABYV, JQ700308; beet chlorosis virus, BChV, AF352024) to 97.0% (TV2) in ORF1-ORF2, 56.7% (ScYLV, AF157029) to 95.2% (turnip yellows virus, TuYV, NC_003743) in ORF3, 50.6% (pepper vein yellows virus 1, PeVYV-1, NC_015050) to 91.3% (TuYV) in ORF4, 50.0% (ScYLV) to 78.1% (beet mild yellowing virus, BMYV, X83110) in ORF3-ORF5; and had aa sequence identity of 16.5% (ScYLV) to 97.6% (TV2) in ORF0 with other poleroviruses, 26.2% (BChV) to 96.1% (TV2) in ORF1, 40.1% (SABYV) to 98.1% (TV2) in ORF1-ORF2, 43.0% (ScYLV) to 94.1% (TuYV) in ORF3, 29.2% (ScYLV) to 88.0% (TuYV) in ORF4, 31.3% (ScYLV) to 77.7% (beet western yellows virus, BWYV, AF473561) in ORF3-ORF5, respectively (Table 3).

**Table 2:** Nucleotide sequence comparisons using the nearly full-length genomic sequences of TPV1 with that of other poleroviruses*.*

Genus name	virus	Sequence identity (%)	GenBank accession numbers
*Polerovirus*	TV2	79.1*	KY038943
	PLRV	67.7	NC_001747
	TuYV	64.4	NC_003743
	BMYV	63.6	X83110
	BWYV	63.3	AF473561
	BrYV	62.7	HQ388348
	BChV	61.6	AF352024
	CYDV-RPS	56.1	NC_002198
	CtRLV	55.5	AY695933
	CABYV	54.9	X76931
	MABYV	54.8	EU000534
	CYDV-RPV	54.7	L25299
	TVDV	54.4	EF529624
	CpCSV	53.9	AY956384
	PYLCV	53.0	HM439608
	MYDV-RMV	52.8	KC921392
	PeVYV-1	52.1	NC_015050
	SABYV	51.6	JQ700308
	ScYLV	**50.4**^**#**^	AF157029

The bold in the table represent the highest and lowest identities individuals.

*The virus shares the highest sequence identity with TPV1; ^#^the virus shares the lowest sequence identity with TPV1.

**Table 3:** Nucleotide and amino acid sequence comparisons of different ORFs of TPV1 with those of other poleroviruses.

Virus name	nt and aa sequence identity between TPV1 and other poleroviruses (%)											
	ORF0		ORF1		ORF1-ORF2		ORF3		ORF4		ORF3-ORF5	
	nt	aa	nt	aa	nt	aa	nt	aa	nt	aa	nt	aa
BChV	49.8	22.7	48.7	**26.2**^**#**^	**54.7**^**#**^	42.3	93.1	91.6	88.8	84.2	77.6	76.7
BMYV	50.6	23.0	51.6	30.9	57.0	45.8	92.1	89.6	88.6	83.6	78.1*	77.4
BWYV	48.2	20.5	52.7	32.3	57.8	46.4	91.8	88.6	88.0	83.6	77.7	77.7*
CtLRV	52.6	22.4	53.7	31.2	60.1	47.6	59.8	52.2	56.8	37.7	55.3	41.5
CYDV-RPS	50.8	27.6	52.1	32.7	58.4	46.9	65.9	63.9	55.5	42.6	57.9	51.1
CYDV-RPV	47.3	23.6	52.2	34.2	58.2	47.6	65.3	65.0	55.2	40.4	57.1	51.1
CpCSV	47.9	19.1	51.0	28.2	57.1	43.0	70.5	70.4	66.8	47.9	56.1	40.2
CABYV	49.7	24.1	51.6	29.4	57.6	44.9	69.5	69.5	65.0	45.5	58.2	48.7
MABYV	49.9	19.4	51.7	30.9	57.9	46.9	71.3	70.1	66.7	47.7	56.3	39.6
PLRV	68.3	54.7	73.2	67.0	77.0	71.1	66.5	62.6	55.4	41.4	56.7	49.4
ScYLV	46.6	**16.5**^**#**^	50.8	28.2	56.7	45.3	**56.7**^**#**^	**43.0**^**#**^	52.5	**29.2**^**#**^	**50.0**^**#**^	**31.3**^**#**^
TVDV	51.8	26.2	50.9	34.4	56.7	48.0	63.3	57.1	53.5	38.5	55.0	44.2
TuYV	48.7	18.6	52.4	36.5	57.7	48.2	95.2*	94.1*	91.3*	88.0*	80.0	80.3
CLRDV	49.7	20.9	52.1	32.7	58.0	48.4	79.3	78.7	75.7	63.4	58.0	48.2
SABYV	45.8	22.0	48.4	27.4	**54.7**^**#**^	**40.1**^**#**^	72.3	70.3	67.8	53.1	55.9	40.4
PeVYV-1	50.9	22.1	51.6	34.8	57.4	47.1	62.1	54.4	**50.6**^**#**^	35.5	52.8	37.8
MYDV-RMV	49.1	20.0	**29.6**^**#**^	30.7	56.6	45.2	65.0	60.0	62.8	38.8	53.1	37.3
BrYV	48.7	19.8	52.7	36.1	57.8	48.3	94.1	92.1	90.2	86.9	77.9	70.3
WYDV-GPV	51.9	29.4	52.5	32.4	58.4	47.1	63.8	59.8	54.0	38.2	57.8	48.5
PYLCV	50.1	24.3	52.1	35.7	57.6	47.6	63.1	55.3	50.9	36.8	53.7	35.4
PABYV	48.0	19.0	48.8	28.3	54.7	40.3	70.8	69.6	66.7	50.0	57.5	48.3
MYMV	45.2	20.6	50.0	30.8	56.0	42.2	66.8	61.3	63.3	38.0	53.0	35.4
TV2	97.3*	97.6*	96.4*	96.1*	97.0*	98.1*	65.9	62.6	54.0	38.5	54.6	49.6
TPV2	**49.2**^**#**^	23.0	52.4	35.8	53.1	45.6	64.2	56.5	54.8	37.7	55.0	44.8

The bold in the table represent the highest and lowest identities individuals.

*nt* Nucleotide, *aa* amino acid, *the virus shares the highest nt or aa sequence identity with TPV1; ^#^the virus shares the lowest nt or aa sequence identitiy with TPV1.

It is worthy to note that the TPV1 ORF5showed the highest differences with that of other poleroviruses. In contrast, the TPV1 ORF3 has the highest nucleotide sequence similarities or the aa sequence identities with that of poleroviruses. The aa sequence identities between the TPV1 P4 or the ORF3-ORF5 readthrough protein and those of other 19 poleroviruses are all less than 90%. The results also showed that TPV1 had the highest nt and aa sequence identity over 96% in ORF0, ORF1 and ORF1-ORF2 with TV2, while had 54.0–65.9% and 38.5–62.6% identity at nt and aa sequence level in ORF3, ORF4 and ORF3-ORF5 with TV2, respectively. With the exception of TV2, TPV1 shared the highest aa sequence identity of 54.7% (ORF0), 67.0% (ORF1), 71.7% (ORF1-ORF2), 94.1% (ORF3), 88% (ORF4) and 77.7% (ORF3-ORF5) with other poleroviruses. In Table 3 we can see that there have high identities between TPV1 and TV2 in 5′ proximal ORFs, TPV1 and TuYV in 3′ proximal ORFs, It is speculated that there may be recombination events in TPV1, TV2 and TuYV. The values are under the current species demarcation criteria for the Solemoviridae^9^, indicating that TPV1 should be a novel species in genus *Polerovirus*.

TPV2 shared the nt sequence identity from 50.2% (SABYV, JQ700308) to 70.4% (TVDV, EF529624) among the 19 poleroviruses (Table 4). The nucleotide and amino acid sequences of different ORFs were compared for TPV1 with 23 selected polervirouses, the results revealed that TPV1 shared nt sequence identity of 43.3% (ScYLV, AF157029) to 64.1% (PYLCV, HM439608) in ORF0 with other poleroviruses, 41.9% (PLRV, NC_001747) to 67.6% (PYLCV, HM439608) in ORF1, 50.1% (SABYV, JQ700308 and CYDV-RPV, L25299) to 71.0% (PeVYV-1, NC_015050) in ORF1-ORF2, 55.7% (ScYLV, AF157029) to 92.3% (PeVYV-1, NC_015050) in ORF3, 50.5% (CtLRV, AY695933) to 93.4% (PeVYV-1, NC_015050) in ORF4, 50.0% (MYDV-RMV, KC921392) to 72.0% (TVDV, EF529624) in ORF3-ORF5, respectively (Table 5). In Table 5 we can see that there have high identities between TPV2 and PeVYV-1 in 3′ proximal ORFs. It is speculated that there may be recombination events in TPV2 and PeVYV-1. The values are also under the current species demarcation criteria for the Solemoviridae^9^, suggesting that TPV2 could be a distinct member in genus *Polerovirus*.

**Table 4:** Nucleotide sequence comparisons using the near full genomic sequence of TPV2 with that of other poleroviruses*.*

	Virus	Sequence identities (%)	GenBank accession numbers
*Polerovirus*	TVDV	70.4*	EF529624
	PeVYV-1	67.9	NC_015050
	PYLCV	66.5	HM439608
	PLRV	57.0	NC_001747
	TV2	56.6	KY038943
	TuYV	55.7	NC_003743
	BrYV	55.2	HQ388348
	BWYV	55.1	AF473561
	BMYV	54.1	X83110
	CABYV	54.1	X76931
	BChV	52.9	AF352024
	MABYV	52.8	EU000534
	CtRLV	52.7	AY695933
	CpCSV	52.5	AY956384
	CYDV-RPS	52.4	NC_002198
	MYDV-RMV	52.4	KC921392
	ScYLV	51.0	AF157029
	CYDV-RPV	50.8	L25299
	SABYV	**50.2**^**#**^	JQ700308

The bold in the table represent the highest and lowest identities individuals.

*The virus shares the highest sequence identities with TPV2; ^#^the virus shares the lowest sequence identities with TPV2.

**Table 5:** Nucleotide and amino acid sequence comparisons of different ORFs of TPV2 with those of other poleroviruses.

Virus name	nt and aa sequence identity between TPV2 and other poleroviruses (%)											
	ORF0		ORF1		ORF1-ORF2		ORF3		ORF4		ORF3-ORF5	
	nt	aa	nt	aa	nt	aa	nt	aa	nt	aa	nt	aa
BChV	47.0	21.2	47.2	28.4	51.2	44.0	63.4	55.1	56.4	40.3	55.4	44.0
BMYV	48.8	29.3	49.2	34.9	53.8	48.9	63.3	55.1	57.5	42.1	55.4	45.0
BWYV	47.3	31.0	51.2	34.4	56.2	49.9	63.5	55.6	56.6	41.6	55.5	44.5
CtLRV	45.1	23.2	48.3	35.7	53.9	49.9	57.7	48.8	**50.5**^**#**^	**25.9**^**#**^	54.2	40.2
CYDV-RPS	46.1	23.4	50.1	31	52.2	44.1	63.6	58.2	62.9	45.0	51.3	41.2
CYDV-RPV	43.6	21.0	47.3	29.3	**50.1**^**#**^	44.2	64.0	58.2	64.2	46.9	51.9	42.4
CpCSV	47.0	20.4	49.2	32.4	53.6	46.1	61.6	54.9	51.9	34.0	52.1	35.3
CABYV	47.2	31.9	50.6	34.3	55.5	49.5	66.2	57.3	54.2	38.6	51.9	37.4
MABYV	44.4	29.6	48.5	33.1	54.7	49.5	64.4	58.3	54.3	38.1	51.1	35.1
PLRV	43.5	20.9	**41.9**^**#**^	31.4	53.3	45.8	63.4	53.6	62.1	43.3	63.0	58.4
ScYLV	**43.3**^**#**^	21.3	50.9	32.4	53.0	**41**^**#**^	**55.7**^**#**^	**44.7**^**#**^	53.0	28.9	50.6	**31.7**^**#**^
TVDV	62.6	49.4*	66.6	58.2	70.9	70.4*	89.7	89.3	90.7	84.0	72.0*	64.8*
TuYV	48.5	27.7	51.8	37.0	57.2	53.0	63.7	56.3	57.0	42.0	54.8	45.1
CLRDV	45.1	23.2	48.3	35.7	53.9	49.9	62.8	59.4	56.9	38.7	52.6	37.8
SABYV	48.1	**18.5**^**#**^	46.8	**28.1**^**#**^	**50.1**^**#**^	41.1	65.2	58.3	54.9	36.5	51.7	34.4
PeVYV-1	63.2	47.6	67.9	59.6	71.0*	68.8	92.3*	91.3*	93.4*	87.8*	66.1	58.0
MYDV-RMV	46.9	22.3	49.1	34.4	54.2	48.0	61.4	52.4	52.7	34.5	**50.0**^**#**^	32.9
BrYV	45.3	25.9	49.7	38.4	55.7	53.7	62.7	57.3	55.8	39.3	55.2	44.6
WYDV-GPV	46.6	24.8	48.6	31.0	51.7	44.2	63.5	54.6	63.8	44.3	53.9	40.2
PYLCV	64.1*	48.8	67.6*	59.9*	70.6	56.3	90.3	89.3	89.8	85.9	59.7	43.8
PABYV	**47.6**	21.0	**51.1**	**29.9**	53.5	43.5	66.5	56.9	55.0	37.1	53.0	38.4
MYMV	**44.2**	20.9	**51.8**	**34.0**	55.7	48.4	61.1	52.4	51.1	29.9	50.5	32.0
TV2	47.4	22.3	50.8	35.6	53.7	45.6	60.3	51.9	59.8	42.7	60.5	57.1

The bold in the table represent the highest and lowest identities individuals.

*The virus shares the highest sequence identity with TPV2; ^#^the virus shares the lowest sequence identity with TPV2.

**Figure 3:**
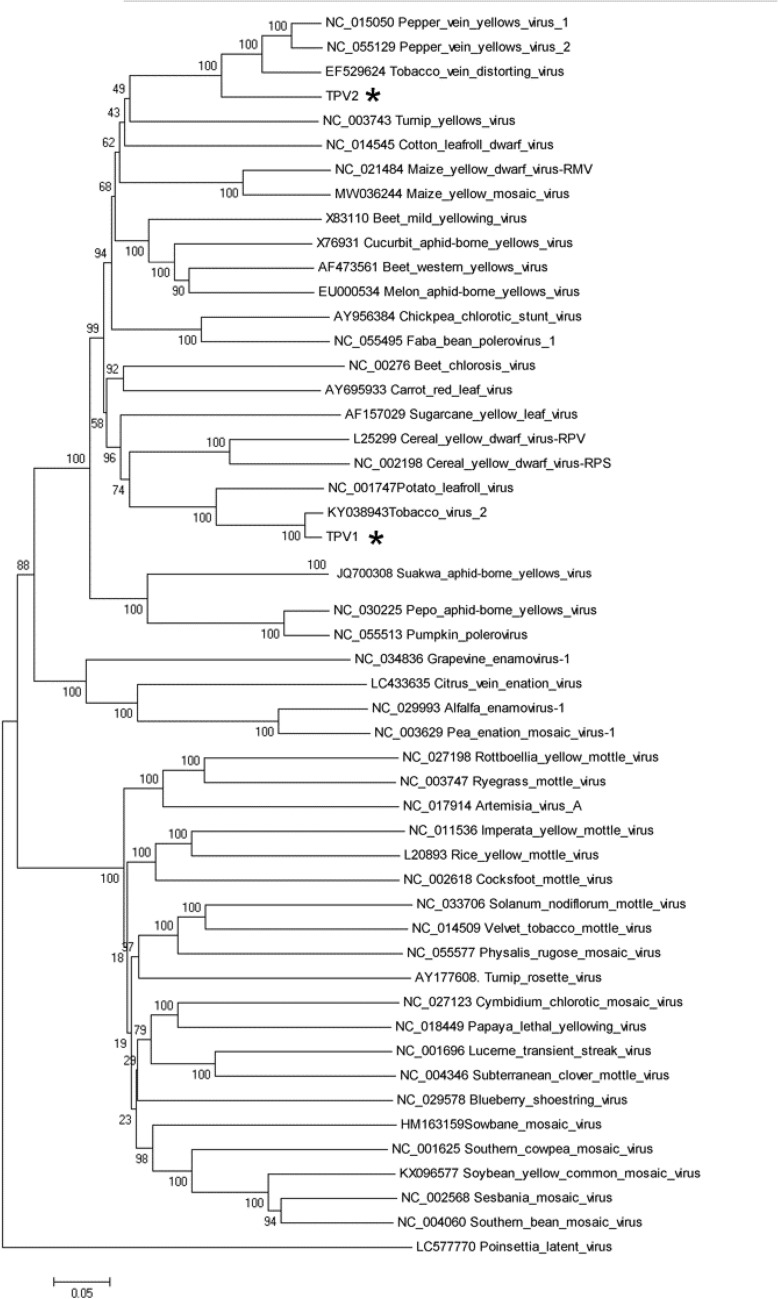
Neighbor joining tree (NJ) of the RdRp nt sequences of TPV1, TPV2 as well as those of other viruses in the family Solemoviridae. The phylogenetic trees are based on alignments of the nt sequences. The sequences were aligned with Clustal W and NJ trees constructed with MEGA 5.0. The scale bar indicates the genetic distance. *Two novel poleroviruses.

To determine the phylogeny among TPV1, TPV2 and thirty representative viruses in the 4 genera of family Solemoviridae, phylogenetic tree was constructed with their RdRp nt sequences by MEGA 5.0 program. The results showed that TPV1 and TPV2 can be clustered with 56 members of family Solemoviridae (Fig. 3). TPV1 is closely related to TV2 and potato leafroll virus (PLRV), while TPV2 is closely related to TVDV, PeVYV-1 and PeVYV-2. The phylogenetic analysis results further revealed that TPV1 and TPV2 are two distinct new poleroviruses.

### Survey of the TPV1 and TPV2 infections in field tobacco plants

To verify the occurrence of TPV1 and TPV2 in field, 244 leaf samples were randomly selected from 817 virus-like tobacco fields samples collected in 2013 to 2018 in Yunnan Province, and tested for TPV1 and TPV2 infections through RT-PCR. The results showed that 8 samples were single infected with TPV1 (detection rate of 3.28%) and 59 samples were single infected with TPV2 (24.18%) (Table 6). In addition, 13 samples were infected with both TPV1 and TPV2 (5.33%). The average detection rate of TPV 1 or TPV2 were up to 32.79%, suggesting that TPV1 and TPV2 were common on tobacco these years.

**Table 6:** RT-PCR detection results for TPV1 and TPV2 from 244 field tobacco samples.

Detected pathogens	Number of diseased samples/detection rate (%)
TPV1 single infected	8/3.28
TPV2 single infected	59/24.18
TPV1 and TPV2 co-infected	13/5.33
Neither TPV1 nor TPV2	164/67.21
Total samples	244

Then 33 TPV1, TPV2, or TPV1 + TPV2 infecting samples were selected and tested for TBTV, TVDV, TBTVsatRNA and TVDVaRNA infections by RT-PCR with virus specific primers. The results showed that TPV1 and TPV2 always co-infected field plants with two to four TBTD casual viruses (Table 7). For example, five samples were co-infected with all six viruses, and 11 samples were co-infected with five different viruses. No single TPV1 or TPV2 infection was detected, and TPV1 or TPV2 always co-infected with both TVDV and TVDVaRNA. It’s speculated that TPV1, TPV2 may have a synergistic relationship with the causal agents of TBTD, and the interactions among TPV1, TPV2 and the causal agents of TBTD is also worthy for further study.

**Table 7:** RT-PCR detection of TPV1, TPV2 and the four TBTD causal viruses in 33 field collected tobacco samples.

Combination of viruses	Number of positive samples	Detection rate (%)
TVDV + TVDVaRNA + TPV1	2	6.06
TVDV + TVDVaRNA + TPV2	5	15.15
TVDV + TVDVaRNA + TPV1 + TPV2	5	15.15
TVDV + TVDVaRNA + TBTVsatRNA + TPV1	2	6.06
TVDV + TVDVaRNA + TBTVsatRNA + TPV2	3	9.09
TVDV + TVDVaRNA + TBTVsatRNA + TBTV + TPV1	3	9.09
TVDV + TVDVaRNA + TBTVsatRNA + TBTV + TPV2	4	12.12
TVDV + TVDVaRNA + TBTVsatRNA + TPV1 + TPV2	4	12.12
TVDV + TVDVaRNA + TBTVsatRNA + TBTV + TPV1 + TPV2	5	15.15
Total	33	

### Survey of the TBTV, TVDV, TBTVsatRNA and TVDVaRNA infections in different field plants

To survey the occurrence of TBTV, TVDV, TBTVsatRNA and TVDVaRNA in field plants, 817 tobacco leaf samples and 733 leaf samples from plants belonging to 29 plant species were collected in Yunnan Province from 2013 to 2018 (Supplementary Table 1). In addition, 65 pepper leaf samples from nine provinces/autonomous regions, and 83 tomato leaf samples from eight provinces/autonomous regions of China were also collected. All the sampled plants showed virus-like symptoms. Eleven crofton weed leaf samples were also collected from Guizhou, 1 purple perilla and 3 dahlia were collected from Liaoning. These collected samples were then tested for TBTV, TVDV, TBTVsatRNA and TVDVaRNA infections through RT-PCR with virus specific primers as described by Liu et al. in 2014. The results showed that 22 plant species in 12 families of Asteraceae (Crofton weed, Dahlia and Sticktight), Solanaceae (Black Nightshade, Potato, Pepper, Tomato, Tobacco), Fabaceae (Broad bean, Pea, Kidney bean), Brassicaceae (Brassica pekinensis, Radish, Oilseed rape), Cueurbitaceae (Pumpkin), Caricaceae (Papaya), Poaceae (Wheat), Araceae (*Amorphophallus konjac*), Araliaceae (Sanqi), Dioscoreaceae (Yam), Liliaceae (Garlic) and Amaranthaceae (Alligator weed) were infected with at least one of the four assayed viruses (Table 8). Among the virus infection 12 families, family Fabaceae, Brassicaceae, Cueurbitaceae, Caricaceae, Poaceae, Araceae, Araliaceae, Dioscoreaceae, Liliaceae and Amaranthaceae had not been reported as the hosts of TBTV, TVDV, TBTVsatRNA and TVDVaRNA. In this study, sticktight, broad bean, pea, oilseed rape, pumpkin, tomato, crofton weed and black nightshade plants were firstly found to be infected with all four assayed viruses of TBTV, TVDV, TBTVsatRNA and TVDVaRNA (Table 8). The presence of the causal agents of TBTD were firstly confirmed in Guizhou, Hainan, Henan, Liaoning, Inner Mongolia and Tibet besides Yunnan. These results suggest that the causal agents of TBTD were widely distributed in China and have spread to a broader plant hosts.

**Table 8:** Detection of the four TBTD causal viruses in 29 species of plants collected from 11 different provinces or autonomous regions in China.

Plant		Pathogens				Sampling areas
Family	Species	TBTV	TBTVsatRNA	TVDV	TVDVaRNA	
Asteraceae	Crofton weed	+	+	+	+	Guizhou, Yunnan
	Dahlia	+	−	−	−	Liaoning
	Sticktight	+	+	+	+	Yunnan
Solanaceae	Black Nightshade	+	+	+	+	Yunnan
	Potato	−	−	+	−	Yunnan
	Pepper	+	−	+	+	Yunnan, Henan, Tibet, Hainan, Liaoning
	Tomato	+	+	+	+	Yunnan, Hainan, Tibet, Inner mongolia
	Tobacco	+	+	+	+	Yunnan
Fabaceae	Broad bean	+	+	+	+	Yunnan
	Pea	+	+	+	+	
	Kidney bean	+	+	+	–	Yunnan
	Abrus precatorius	−	−	−	−	
	Soybean	−	−	−	−	
Brassicaceae	Brassica pekinensis	−	−	+	+	Yunnan
	Radish	−	−	+	+	
	Oilseed rape	+	+	+	+	
Cucurbitaceae	Pumpkin	+	+	+	+	Yunnan
	Cucurbit	−	−	−	−	
	Wax gourd	−	−	−	−	
	Cucumber	−	−	−	−	
Caricaceae	Papaya	−	−	+	−	Yunnan
Poaceae	Wheat	−	−	+	+	Yunnan
Araceae	Amorphophallus konjac	+	+	+	−	Yunnan
Araliaceae	Sanqi	−	−	+	−	Yunnan
Dioscoreaceae	Yam	−	−	+	−	Yunnan
Lamiaceae	Purple Perilla	−	−	−	−	Yunnan
	Red sage	−	−	−	−	
Liliaceae	Garlic	−	−	+	−	Yunnan
Amaranthaceae	Alligator weed	−	−	+	−	Yunnan

+ Positive detection, − negative detection.

**Figure 4:**
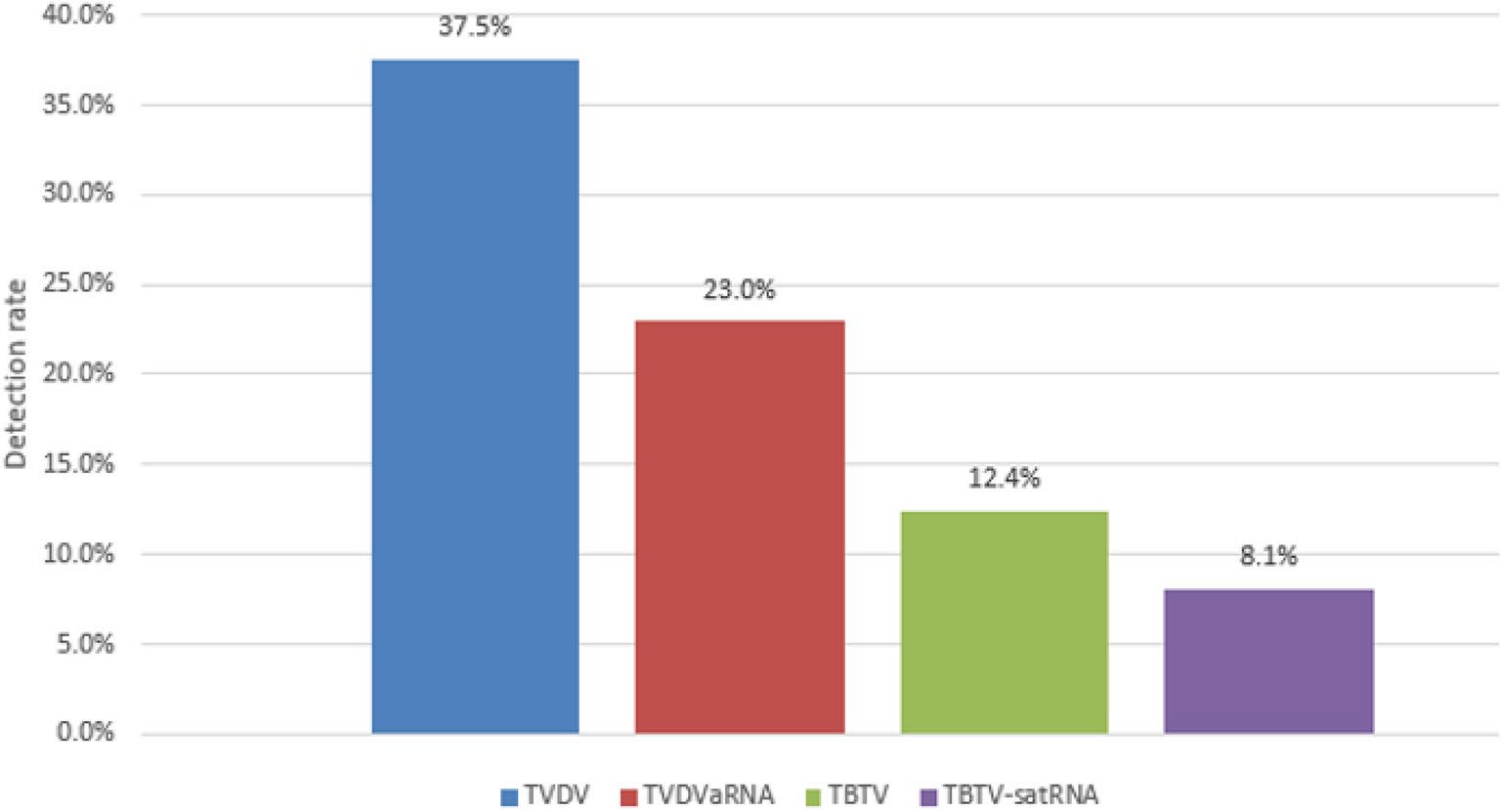
Detection rate of TBTV, TVDV, TBTVsatRNA and TVDVaRNA in field collected samples.

In this study, 663 out of the 1713 tested leaf samples were found to be infected with TBTV, TVDV, TBTVsatRNA and/or TVDVaRNA, with the average detection rate of 38.70% (Table 9). The result also showed that, among the four assayed viruses, the infection rate of TVDV was the highest (37.5%) while the infection rate of TBTVsatRNA was the lowest (8.1%) (Fig. 4). Six hundred and sixty-three samples were detected at least one causal agents of TBTD. It was found that the combination of the two to four causal agents of TBTD in the field was commonly. Meanwhile, there are 364 samples co-infected with two causal agents of TBTD (TBTV + TVDV, TBTV + TBTVsatRNA or TVDV + TVDVaRNA) (accounting for 54.9% of these 663 TBTD diseased samples) . Fivty-one samples co-infected with 3 causal agents of TBTD combined with TBTV + TVDV+TVDVaRNA, TBTV+TVDV + TBTVsatRNA (accounting for 7.7% of these 663 TBTD diseased samples), 86 samples co-infected with all four causal agents of TBTD (accounting for 13.0% of these 663 TBTD diseased samples). The causal agents of TBTD were mainly in 2 agents combination in the field, more common for 3 or 4 agents co-infections. TVDV was found in most of the pathogen combinations, which indicated that TVDV played an important role in the occurrence of TBTD in the field. In this study, 156 samples were single infected with TVDV (9.11%) and 6 samples were single infected with TBTV (0.35%), whereas no single TBTVsatRNA or TVDVaRNA infection was detected. TVDV was found in most of the virus combinations indicating that TVDV plays an very important role in the occurrence of TBTD in the field. There were 21 samples infected by TBTV or TBTVsatRNA but ansence TVDV, which declared that there may be other viral agents that can asist TBTV and TBTVsatRNA complete vector transmission.

**Table 9:** Detection of the four TBTD causal viruses in field collected samples.

Viruses	Positive numbers	Detection rate (%)
TVDV	156	9.11
TBTV	6	0.35
TBTV + TBTVsatRNA	15	0.88
TBTV + TVDV	55	3.21
TVDV + TVDVaRNA	294	17.16
TVDV + TVDVaRNA + TBTV	14	0.82
TVDV + TBTV + TBTVsatRNA	37	2.16
TVDV + TVDVaRNA + TBTV + TBTVsatRNA	86	5.02
Negative samples	1050	61.30
Total number of samples/average detection rate	1713	38.70

## Discussion

Several viral agents have been reported to cause TBTD in some countries. For example, an early study had suggested that TBTD in Zimbabwe was caused by a co-infection of TVDV and TBTV^1^. Later researches had shown that tobacco plants showing TBTD-like symptoms in the Yunnan Province, China, were infected with TBTV, TVDV, TBTVsatRNA and TVDVaRNA. Through these researches, the full genomes of TBTV, TVDV and TVDVaRNA have been determined^2,3,5^. In 2014, Abraham and colleagues reported that in Ethiopia, tobacco plants showing TBTD-like symptoms were infected with ETBTV, ETBTVsatRNA and PLRV^10^. Considering that the complete nucleotide sequence identity between ETBTV and TBTV is only about 56.4%, and the full nucleotide sequence identity between ETBTVsatRNA and TBTVsatRNA is only about 35.6%, both ETBTV and ETBTVsatRNA are now considered as new virus species. The nucleotide sequence identity between TBTV Zimbabwe A2 isolate and ETBTV is about 94.9%, these two viruses are now considered as two different isolates of ETBTV. Whether TBTV and TVDV co-infect tobacco in Zimbabwe and Ethiopia remains unclear. Based on the current knowledge, the TBTD found in China and the TBTD found in Africa are two different diseases, and are caused by different causal viruses. Although six different causal viruses have now been found in TBTD-symptoms plants in China, how these viruses co-infect field crops and if more unidentified virus(es) are associated with this disease in field are still unknown. With the development of HTS and bioinformatics in recent years, discovery of new TBTD causal virus(es) or TBTD associated causal agents becomes possible^21,22^. In virus infections plants, large numbers of virus-derived siRNAs (vsiRNAs) will be generated along with the viral genomic RNAs, and these vsiRNAs can be identified and assembled into virus contigs or even full-length viral genome^23–25^. This technology can also help us to identify new virus(es) associated with TBTD.

In this study, HTS was used to analyze two tobacco samples showing typical TBTD-like symptoms in two different locations. Based on the assembled sequences, we have determined two new near full-length polerovirus (i.e., TPV1 and TPV2) sequences. Sequence alignment result showed that TPV1 shares the highest nucleotide sequence identity with TV2 (79.1%). The deduced amino acid sequences of the TPV1 P4 protein and the read-through protein (P3-P5) share less than 90% identities with that of viruses in the genus *Polerovirus*. Sequence alignment result also showed that TPV2 shares the highest nucleotide sequence identity of 70.4% with TVDV. The predicted amino acid sequences of TPV2 proteins, except P3, share less than 90% identities with that of viruses in the family Solemoviridae. Therefor, we conclude that TPV1 and TPV2 are two novel poleroviruses.

Ethiopian tobacco bushy top disease symptoms are similar to that of TBTD in China, and is also caused by several different polerovirus and umbravirus^10^. Recent study showed that ETBTV can complete its vector transmission assist by cowpea polerovirus 1 (genus Polerovirus) besides PLRV^10,26^, and the results of our group also proved that TBTV can complete its aphid transmission with the assistance of barley yellow dwarf virus GAV (genus *Luteovirus*; unpublished data). It can be inferred that there may be other polerovirus could assist TBTV acomplish its aphid transmission. Our survey results also revealed that 0.35% and 0.88% field samples infected TBTV or TBTV + TBTVsatRNA do not coinfected with TVDV (Table 9). That means there could be another polerovirus other than TVDV could help TBTV acomplish its aphid transmission, and TPV1 and/or TPV2 should be a potential aphid transmission help virus for TBTV in nature. The phylogenetic analysis showed that TPV1 is closely related to PLRV and TV2, and TPV2 is closely related to TVDV. Because TPV1 and TPV2 are often co-infected with one or more of the other four TBTD known causal viruses, we speculate that both TPV1 and TPV2 might have important roles in the induction of TBTD-like symptoms and/or in the TBTD disease cycle. However, whether TPV1 and TPV2 are responsible for TBTD in China is remain unknown.

Earlier studies on TBTD were done mainly in tobacco plants in Yunnan Province, China. In recent years, several reports have indicated that TVDV and TBTV can also infect other plant species, including pepper, Dahlia, tomato and crfton weed^16–18^. In this study, we have determined that except tobacco, a total of 21 plant species in 12 families can be infected with at least one of the TBTD causal viruses. In addition, we have found TVDV + TVDVaRNA + TBTV + TBTVsatRNA co-infection in crofton weed in Guizhou Province, tomato plants in Hainan Province, and tobacco, sticktight, broad bean, pea, oilseed rape, pumpkin, tomato as well as black nightshade plants in Yunnan Province, respectively. This new finding indicates that the TBTD causal viruses can infect weeds and many other cash crops, resulting in a large virus reservoir in nature. It is noteworthy that many plants co-infected with these four causal viruses did not show typical TBTD-like symptoms. Taken together, we conclude that the causal agents of TBTD are a group of different viruses, these viruses have much broader host ranges and distributions than what have been reported, and these infected plants can serve as the key overwintering or intermediate hosts for the six causal viruses. These three points should be considered when developing an effective control strategy for TBTD in fields.

## Supplementary Information

Supplementary Information 1.

Supplementary Figures.

Supplementary Tables.

Supplementary Information 2.
