# Supplementary figures and images for: Identification of two novel poleroviruses and the occurrence of Tobacco bushy top disease causal agents in natural plants
Source: Sci Rep. 2021 Oct 26;11:21045. doi: 10.1038/s41598-021-99320-x (PMC8548504; doi:10.1038/s41598-021-99320-x)

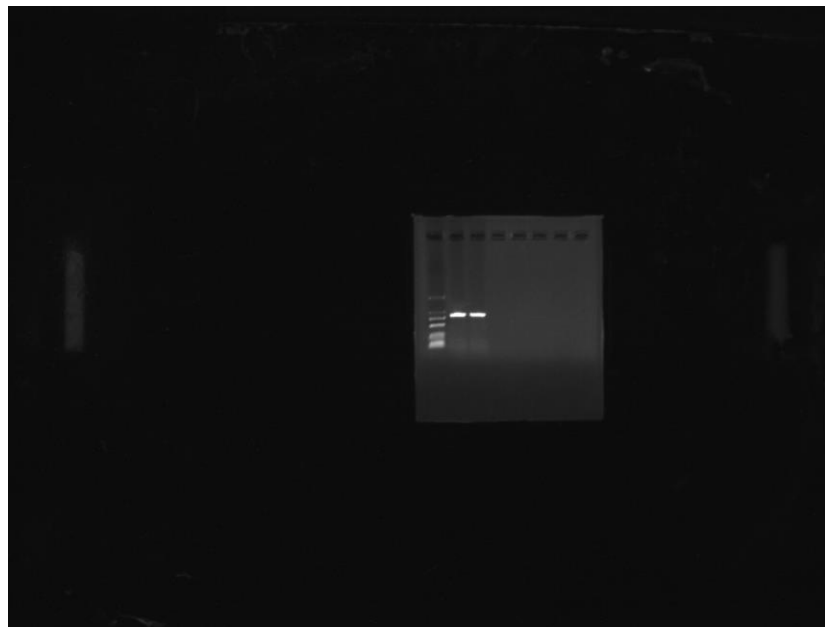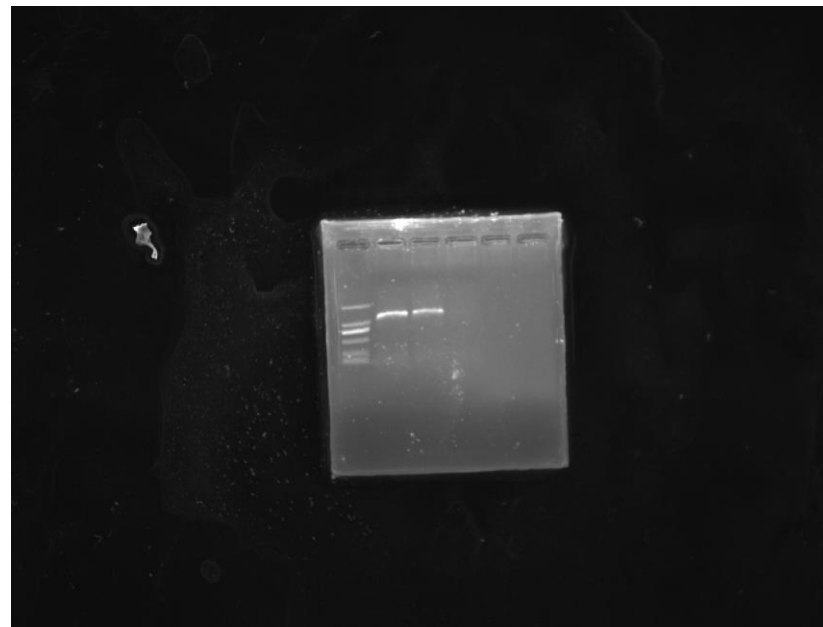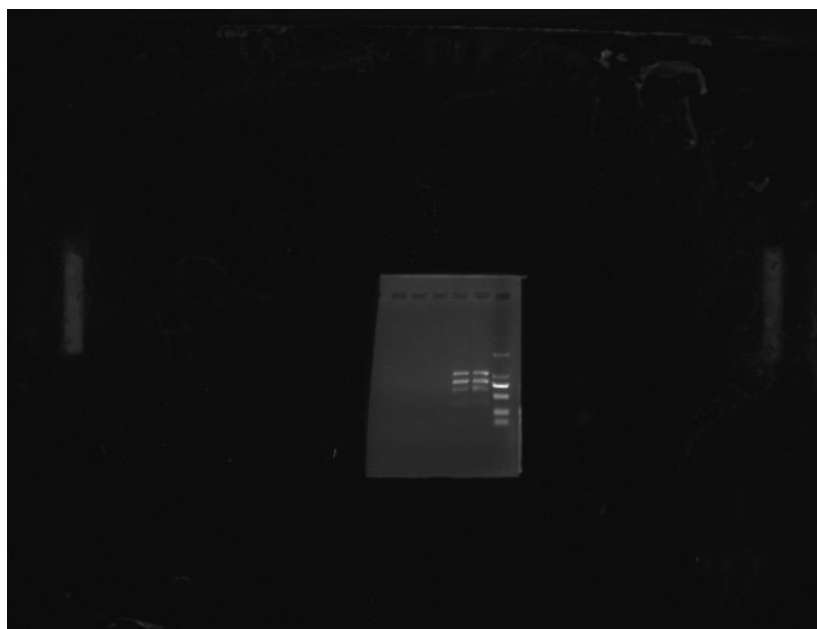

Supplement: Supplementary file 1 — Supplementary Information 1. [file 41598_2021_99320_MOESM1_ESM.pdf]
