# Supplementary material for: Identification of two novel poleroviruses and the occurrence of Tobacco bushy top disease causal agents in natural plants
Source: Sci Rep. 2021 Oct 26;11:21045. doi: 10.1038/s41598-021-99320-x (PMC8548504; doi:10.1038/s41598-021-99320-x)
Supplement: Supplementary file 2 — Supplementary Figures. [file 41598_2021_99320_MOESM2_ESM.doc]

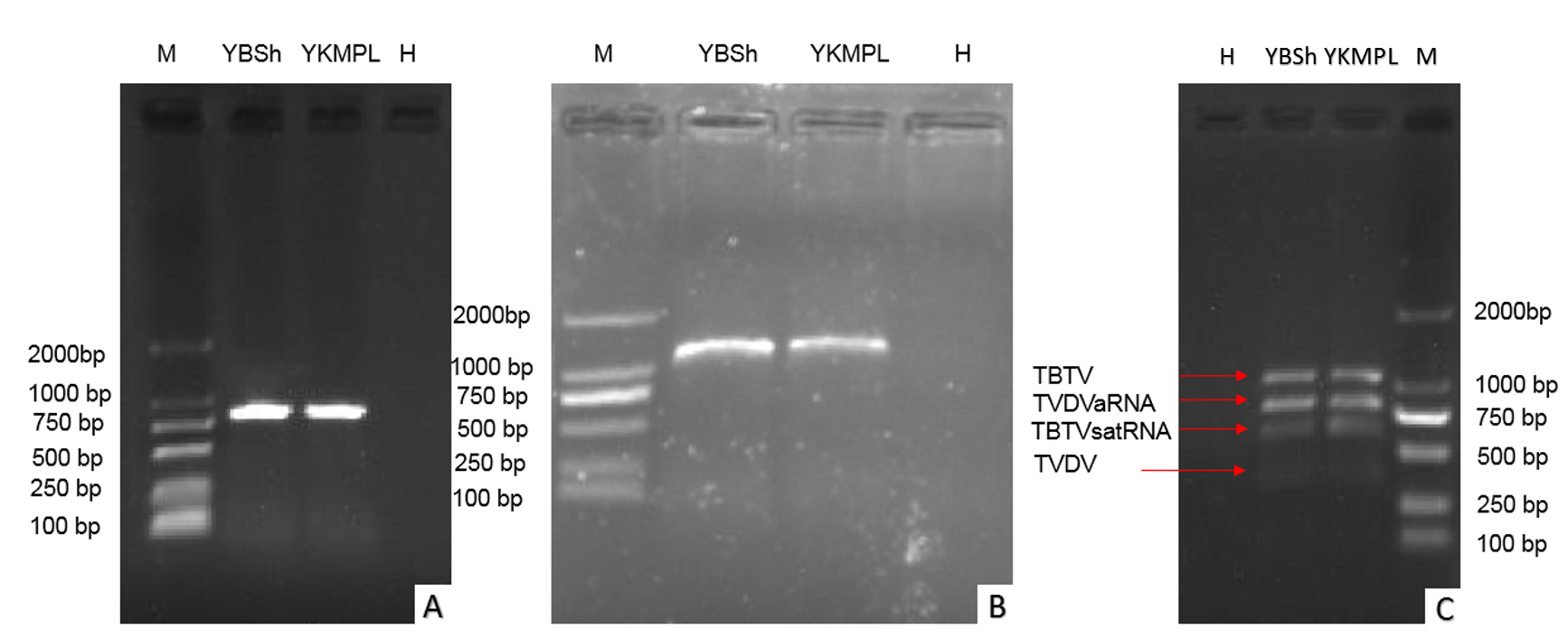


Figure S1. Detection of TPV1, TPV2, TBTV, TVDV, TBTVsatRNA and TVDVaRNA from YBSh and YKMPL samples. TPV1 (A) and TPV2 (B) were detected by conventional RT-PCR respectively. TBTV, TVDV, TBTVsatRNA and TVDVaRNA were detected by multiplex one-step RT-PCR (C), specific virus amplicons are indicated with arrows. H: means healthy tobacco plant, YBSh and YKMPL indicate the two TBTD samples.


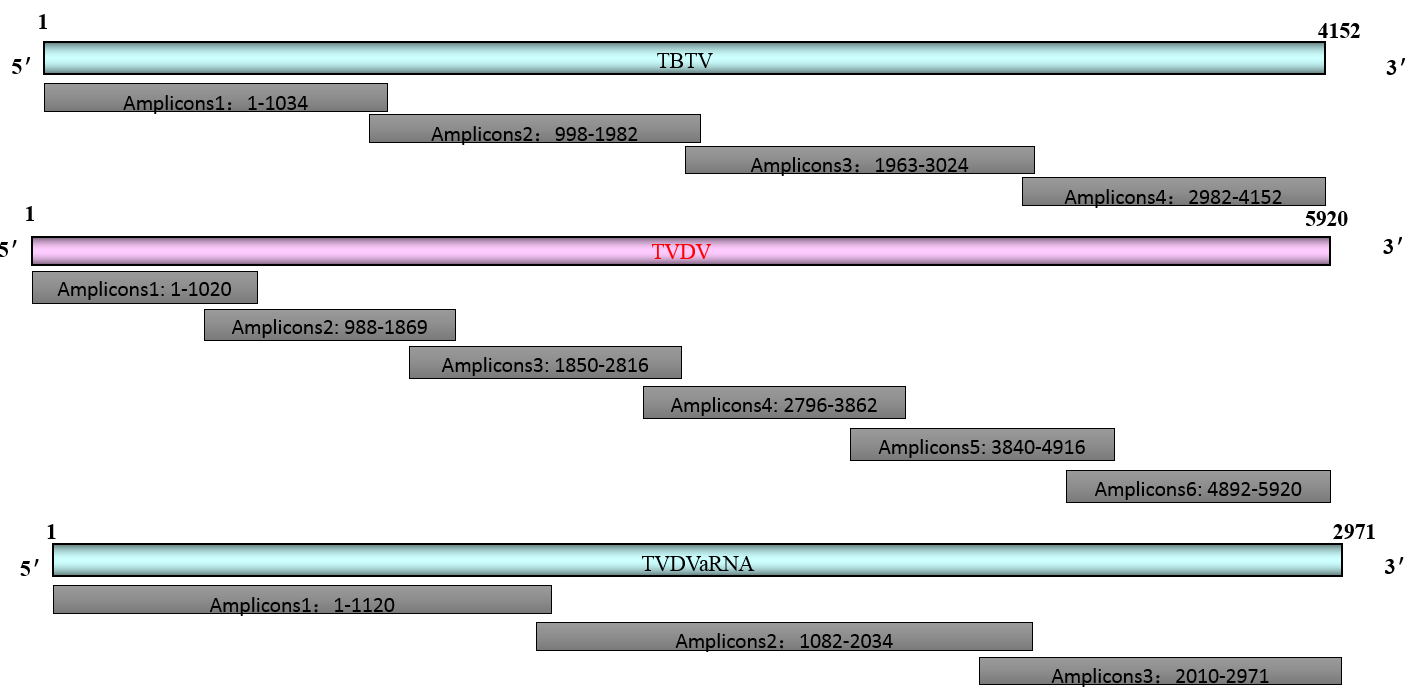


Figure S2. Amplification strategies for TBTV, TVDV and TVDVaRNA genomes.
