# Supplementary material for: Identification of two novel poleroviruses and the occurrence of Tobacco bushy top disease causal agents in natural plants
Source: Sci Rep. 2021 Oct 26;11:21045. doi: 10.1038/s41598-021-99320-x (PMC8548504; doi:10.1038/s41598-021-99320-x)
Supplement: Supplementary file 3 — Supplementary Tables. [file 41598_2021_99320_MOESM3_ESM.docx]

| **Sampling locations** | **Plant species** | | | | | | | | | | | | | | | | | | | | | | | | | | | | | **Sampling number** |
| --- | --- | --- | --- | --- | --- | --- | --- | --- | --- | --- | --- | --- | --- | --- | --- | --- | --- | --- | --- | --- | --- | --- | --- | --- | --- | --- | --- | --- | --- | --- |
|  | **Tobacco** | **Pepper** | **Tomato** | **Potato** | **Black Nightshade** | **Broad bean** | **Pea** | **Kidney bean** | **Azuki bean** | **Soybean** | **Chinese cabbage** | **Radish** | **Oilseed rape** | **Pumpkin** | **Cucurbit** | **Cucumber** | **Wax gourd** | **Papaya** | **Garlic** | **Wheat** | **Sanqi** | **Yam** | **Red sage** | **Purple perilla** | **Konjak** | **Crofton weed** | **Sticktight** | **Dahlia** | **Alligator weed** |  |
| Yunnan | 817 | 96 | 39 | 12 | 12 | 224 | 57 | 20 | 4 | 26 | 5 | 3 | 42 | 35 | 4 | 5 | 12 | 1 | 1 | 5 | 14 | 56 | 1 | - | 13 | 6 | 30 | - | 10 | 1550 |
| Guizhou | - | 17 | - | - | - | - | - | - | - | - | - | - | - | - | - | - | - | - | - | - | - | - | - | - | - | 11 | - | - | - | 28 |
| Liaoning | - | 3 | 7 | - | - | - | - | - | - | - | - | - | - | - | - | - | - | - | - | - | - | - | - | 1 | - | - | - | 3 | - | 14 |
| Henan | - | 11 | 3 | - | - | - | - | - | - | - | - | - | - | - | - | - | - | - | - | - | - | - | - | - | - | - | - | - | - | 14 |
| Tibet | - | 4 | 3 | - | - | - | - | - | - | - | - | - | - | - | - | - | - | - | - | - | - | - | - | - | - | - | - | - | - | 7 |
| Hainan | - | 2 | 25 | - | - | - | - | - | - | - | - | - | - | - | - | - | - | - | - | - | - | - | - | - | - | - | - | - | - | 27 |
| Inner Mongolia | - | 3 | 21 | - | - | - | - | - | - | - | - | - | - | - | - | - | - | - | - | - | - | - | - | - | - | - | - | - | - | 24 |
| Shandong | - | 4 | 7 | - | - | - | - | - | - | - | - | - | - | - | - | - | - | - | - | - | - | - | - | - | - | - | - | - | - | 11 |
| Shaanxi | - | - | 16 | - | - | - | - | - | - | - | - | - | - | - | - | - | - | - | - | - | - | - | - | - | - | - | - | - | - | 16 |
| Zhejiang | - | 1 | 1 | - | - | - | - | - | - | - | - | - | - | - | - | - | - | - | - | - | - | - | - | - | - | - | - | - | - | 2 |
| Hubei | - | 20 | - | - | - | - | - | - | - | - | - | - | - | - | - | - | - | - | - | - | - | - | - | - | - | - | - | - | - | 20 |
| Total | 817 | 161 | 122 | 12 | 12 | 224 | 57 | 20 | 4 | 26 | 5 | 3 | 42 | 35 | 4 | 5 | 12 | 1 | 1 | 5 | 14 | 56 | 1 | 1 | 13 | 17 | 30 | 3 | 10 | 1713 |

**Supplementary Table 1**. Summary of the sample collections from 11 different Provinces and Autonomous Regions in China.

**Supplementary Table 2**. Primers used for the detection of TBTV, TVDV, TBTVsatRNA, TVDVaRNA, TPV1 and TPV2.

| **Primer name** | **Sequence （5′–3′）** | **PCR product size (bp)** | **Virus** | **Reference** |
| --- | --- | --- | --- | --- |
| TBTVdF | TACCACACCTAAACAGCGTTG | 1049 | TBTV | Liu *et al.,* 2014 |
| TBTVdR | CTCATCTCCCGCTAAGTCAG |  |  |  |
| TVDVdF | GCAACAGCGAGACTTTCATCT | 357 | TVDV | Liu *et al.,* 2014 |
| TVDVdR | CRTTGCCTTTATAGAGCAGCC |  |  |  |
| TBTVsatRNAdF | TGTTGGCCGTGGGCAGCAA | 598 | TBTVsatRNA | Liu *et al.,* 2014 |
| TBTVsatRNAdR | TTCGTATTTGGCTCCGTCGC |  |  |  |
| TVDVaRNAdF | GCGTACTGCGGAAGTCTCAC | 792 | TVDVaRNA | Liu *et al.,* 2014 |
| TVDVaRNAdR | TGTACTGGAGTGCTTCAACCG |  |  |  |
| TPV1dF | GCCACTTCAACCAGGGACAG | 752 | TPV1 | this study |
| TPV1dR | AGAAGCTAAGGAAGAACCCC |  |  |  |
| TPV2dF | CTTCAGCCGAGGTGACCTC | 1186 | TPV2 | this study |
| TPV2dR | ATGTAATTAGAAATCACTTC |  |  |  |

**Supplementary Table3.** Primers used for amplification the gemones of TBTV, TVDV, TBTVsatRNA and TVDVaRNA

| Primer | Sequence (5’-3’) | Product size | Target virus |
| --- | --- | --- | --- |
| TBTVa1F | GGGTTACGATATGGAGTTCAT | 1034 | TBTV |
| TBTVa1R | GCCCTAAGCACTCGGATTTT |  |  |
| TBTVa2F | AGACGAAAATCCACCCCAAAA | 985 |  |
| TBTVa2R | GTTCCCTAAACCAAGGCTCGAC |  |  |
| TBTVa3F | GAGCCTTGGTTTAGGGAACT | 1062 |  |
| TBTVa3R | GAATACCACCACAATCTTCT |  |  |
| TBTVa4F | CACGGGGGGACTTTTATTC | 1171 |  |
| TBTVa4R | GGGCGCGAGAGAGAGTGCTCAT |  |  |
| TBTVsatRNAaF | GGGTATCGATACAAGGAGCAG | 824 | TBTVsatRNA |
| TBTVsatRNAaR | TAAGTAGATCCCATCTAGCCC |  |  |
| TVDVa1F | ACAAAATATAAGAAGGGAGA | 1020 | TVDV |
| TVDVa1R | TGAGTGGTATTTTGTTAGTG |  |  |
| TVDVa2F | CTTCCTCAAAGACCACTAACA | 882 |  |
| TVDVa2R | CTATAGCAGAAAGGTCGATC |  |  |
| TVDVa3F | GATCGACCTTTCTGCTATAGA | 967 |  |
| TVDVa3R | AAGGTACTTCTCCCAGGAGGT |  |  |
| TVDVa4F | TCCCACCTGAGGAAGTGATT | 1067 |  |
| TVDVa4R | CTTGTTGAAGATGAAAGTCT |  |  |
| TVDVa5F | GCGAGACTTTCATCTTCAACAA | 1077 |  |
| TVDVa5R | CTAATTCCATATCTCTATCTG |  |  |
| TVDVa6F | TGGACAGATAGAGATATGGA | 1029 |  |
| TVDVa6R | AGGATTACTCACAGTTTTAAA |  |  |
| TVDVaRNAa1F | GGGGATTCATGGAGACAGCT | 1120 | TVDVaRNA |
| TVDVaRNAa1R | AGAAATTCATCTGGGCTCAT |  |  |
| TVDVaRNAa2F | TAGGTGCAGAGCCTCCCCGAT | 953 |  |
| TVDVaRNAa2R | TTAACATGTGGTACTTCATCA |  |  |
| TVDVaRNAa3F | GGGCTGATGAAGTACCACAT | 962 |  |
| TVDVaRNAa3R | GGGGCGGTGTACCTGGTAAAC |  |  |

**Supplementary Table3.** Abbreviation of poleroviruses used in this study

| Virus Abbreviation | Virus Name | Acc. No. |
| --- | --- | --- |
| PeVYV-1 | Pepper vein yellows virus 1 | NC_015050 |
| PeVYV-2 | Pepper vein yellows virus 2 | NC_055129 |
| TVDV | Tobacco vein distorting virus | EF529624 |
| TuYV | Turnip yellows virus | NC_003743 |
| CtLDV | Cotton leafroll dwarf virus | NC_014545 |
| MYDV-RMV | Maize yellow dwarf virus-RMV | NC_021484 |
| MYMV | Maize yellow mosaic virus | MW036244 |
| BMYV | Beet mild yellowing virus | X83110 |
| CABYV | Cucurbit aphid-borne yellows virus | X76931 |
| BWYV | Beet western yellows virus | AF473561 |
| MABYV | Melon aphid-borne yellows virus | EU000534 |
| CpCSV | Chickpea chlorotic stunt virus | AY956384 |
| FBPV-1 | Faba bean polerovirus 1 | NC_055495 |
| BChV | Beet chlorosis virus | NC_002766 |
| CtRLV | Carrot red leaf virus | AY695933 |
| ScYLV | Sugarcane yellow leaf virus | AF157029 |
| CYDV-RPV | Cereal yellow dwarf virus-RPV | L25299 |
| CYDV-RPS | Cereal yellow dwarf virus-RPS | NC_002198 |
| PLRV | Potato leafroll virus | NC_001747 |
| TV2 | Tobacco virus 2 | KY038943 |
