# Supplementary material for: Identification of two novel poleroviruses and the occurrence of Tobacco bushy top disease causal agents in natural plants
Source: Sci Rep. 2021 Oct 26;11:21045. doi: 10.1038/s41598-021-99320-x (PMC8548504; doi:10.1038/s41598-021-99320-x)
Supplement: Supplementary file 4 — Supplementary Information 2. [file 41598_2021_99320_MOESM4_ESM.pdf]

>SeqID [organism=Tobacco bushy top virus] [moltype=genomic RNA] [isolate=YBSh] tobacco bushy top virus isolate YBSh, complete genome

GGGTTACGATATGGAGTTCATCAACAAGATAAAGCAATTGTTGGCAATGAATTTCAAGCCCTCGAAGGGCGT
AATGTCTCGGGAGGAGCTCCGTGAAGCTTTCGATCCCACTTGGGAGCTCCTCATCACACAGGCTCGTGTCAC
CACTGAGGTGTCACGCCAGTGTGAGGATTGGTACACTCTAGCTGTACCCACTACCTACCGCTTGCCAGAGTT
GGCGGTAGAAGAGGCTGTGCGCGAAAAGAACATAGCGCGAGAGGTGGCGATCAAGTGCCCCCCTGAGGA
CCCGATTCCCGCAACCACGCAGGAGTCACAGCCTTTTCCCCTGGTGAGGAGTGCTGAACGCTCTAGCCAGG
ATGCCGAGCGTGAGGCGCTCGATGAGATATGGGGGCTCCCCACTCCCGCGCCAAATCCTCTGCCCAAGTACT
TCGAGCGGCGCTACCAGGCACTGGCTCGGACCTGCCAGAAGGACTACTCCAACTGGCAGATTGTGCCCTAC
ACGGGTGGGCCACGCGTGCTGAGAGAGGAGGACGTGTTACCAATGGCCACGGGGGTAATACCCTTGCCAT
CACCCCCCCCTAAGGCTTCTGTAATTGGAGCAGTATTGGGGCTGATTACCCGCCTCACCAAGGTGGCGGGG
GAGGTGAAGAGCAGGCTGAGCGTCTCGCCGCGGGAGCCCTCTCCCACCTGCATTGGCTTAGAGCAGGTAG
CTGGTGAGCCCATGGGCTACATGAATGCACACTCTGTAGCTATGGAGCTGCGGGCTAGGTACGGAGTCCAG
CCCGCCACAGCTGCAAACTTGCAGCTTGGAAACCGGGTGGCCAGGGAAATCCTGGAAAAACAGTGCGGG
GCCACCCGCGACATGGTGTTCATACTCGGGCACCTCGCCACCACCTTGTGGTTCACTCCCACCATGGTGGAC
TTGGCCCTTCAATGTGGGCCTAAGGATTTTTGCTAGGGGATGTGGTCGCTCGGAGGGGTGTAGAAACTAAA
GTGAAGACGAAAATCCACCCCAAAATCCGAGTGCTTAGGGCGGCCCGTCCCCGGCCCGTAGAAAGAGTGTC
GTACCAGATCGACGTGGTGCGGCCCTGTGCTGACTTTGGAGTCCACAACAACTCCCTCAACAATTTGGTGCG
AGGGGTTAACGAGCGGGTGTTCTACACAGACCACAAGAGGAAAGAGCCCCGCAGACCTTCAGCTGGTAGT
TTCGACAAGATCGACATCAGCGAGATAAAAGCATTCAGAGTCCAGCCGTGGACTCTCGAGGAGGTCGTCGA
CAGCTACACGGGTAGCCAGAGGGTGCGGTATGGACAAGCTGTTGAATCCTTAGCAGTAACGCCCCTCTCAC
GAAACGACGCCCGGGTCAAAACATTTGTGAAGGCGGAAAAGATAAACTTCACCGCCAAGCCCGACCCGGC
TCCTCGTGTTATCCAGCCGCGGGATCCAAGGTTCAATGCCTGCTTTGCCAAATACACAAAGCCCTTGGAACC
CCTCCTATATAAGCAGCTGGGTAAGCTTTACCAGTTCCCATGCATCGCAAAAGGCTTCAACGCCGTAGAGAC
CGGAGAGATAATAGCCAAGAAGTGGAAGTGCTTCAATGACCCTGTCTGTGTGGGTTTAGATGCCTCCCGGTT
TGACCAACATGTGTCATGCGATGCACTACGGTTCACCCATAGCGTGTACAAACGGTTCGTGAAGGGCAGGG
AAGTGAACAAGTTGCTTTCCTGGATGTACAAGAACCACGCTCTGGGAAGTGCGAAGGACGGATTCGTCAAG
TATGAGGTGGAAGGCTGTCGGATGAGTGGTGATATGGATACGGCCCTAGGGAACTGTGTCCTGATGGTCCTC
ATGACTAGGCAGCTATGCAAAAACCTCTCCATACCGCACGAACTGATGAACAACGGCGATGACTGCATAGTT
ATATTCGACAGGCAGCACCTGTCCACCTTTCAGGATGCAGTCGAGCCTTGGTTTAGGGAACTAGGGTTTACA
ATGAAGGTCGAGGAGCCAGTCTACCATCTCGAAAGAGTAGACTTTTGCCAGACCCGTCCCGTGTACGACGG
CAAGAAGTGGAGAATGGTCAGGCATATCTCAAGTATAGCCAAGGATTGCTGTTCAGTTATTGATTGGGAGCA
GTTGCCGGCTTGGTGGAACGCCATTGGAGAATGTGGCATTGCCGTGGCTGGTGGTATACCCATACACAACA
GCTTCTTGAGATGGCTCCTGAGATCAGGTGAGAGCAACCCTGATCTCCTGAAGCATGGCGCATGGAAAAAT
GAGGGCCTAGCGTGGTACCGGATGGGTATGGACCTATCCCATGAGAGACACGTTAGTGATGAAGCGCGCGC
CAGCTTCCACACTGCCTTTGGAATCGAACCATCCATGCAGGTCGCATTAGAGCAGATCTATGACTCATTGCCT
GCTCCCACCATTGGTGGGAAACGAGCCAGAGTATGTAAACCTGGAGAAATGGTATTGGTTGATTCACTCCCA
CCGCGGCACTTTAATAATTACTTCCAGGATGTTGGAATGGGGGGGAGCAGTAGTGATTACGTTGTCCCGGG
GACCCACGAGTTCGAACCGGGAACGTTGTGGACACAATGCTAGTCGACTGAGTTAGCCAGGCATATGGTTG
CAAGCGCTGGGGACGTAGTCCATAAAGCCACTGGTAAAGAGCCTCCAGTAGGCAGGCCCGCCTTGATGAGT
CTTAACGAGTGGACACCCGGGTAGCCGCGGACAATGCCCGACGGGTTTCAATGTCTACGATCATAAATGTCA
ACACTCATCCACAAGGCAGATACGAAAGAGGAGCTGCTCAACGCTCTATACGGGGAAGTGACGTTAAGGGA
GCTCGAGGAATCAAACCTCGGCGTACTAACCCCCCACCGGGCCGAAAACAAGGTGGTAATGACCCCACTGC
TGCCACCCAGAAGTCAAGGAAGAATCGCCAGCGTCTTGAAACGGTTCCGCCCCACGAAACACACGGGGGG

ACTTTTATTCATAGAGAAGATTGTGGTGGTATTCACGCCCCACATCCCGGACGATGCTGGCGGGGAGGTAGA
AATCTGGGTCCACGACAATATGCTACCACACCTAAACAGCGTTGGGCCCCGCGTAAGATTTCCGATGAGCGG
AGGGCCCAGGTTGATAGCATTTTATCCCCCCTACTCGATACCCTTAAGTTGTCAAGTGAGGGGGGCACCCCG
GAGTTACTTCATTGTATCGGAGTACTCCGGCGTGAACTTCGTCGCAGGCGCGAGCCCGTTCAGCCTGTACAT
AATGTGGGAGCCGAAGATAGAGTGCGTAGCGCACAACTATCTGATGAGGCCCCCCAAAGCAATGCCGATTT
GCAGACATCTGGTAAAGGACAGTCTGTCCTCCCTAACCCTGACCCAGGGGGCACTGAAAAGTGCAATGTCG
AATCGGTATGCAACAACTGCGACCGGGCTCCCACCACCAACAAGTGGTGAGCAGGACATGGAGGTAGTGT
CTCATCCTCCAGGCTAAATTGGACACAAAACGGAGCTGGATTTAGTGTTGCAATTGCCAGACCGTAAGCCAC
TACGCTAGACAGTGCGACAACTGAGGTCGTCTATGACCCACAGGGTGCCATCCTGTGCAATTTCCAACCCTT
GGCAAGGGTGATCCTAGAGGACGCGGGCATTTACTTGCCTGGGGATGCCTCTAGTTACATACCGGAGTGGT
TGATAGACCTCTGTGTAAGGAAACAGGTGTGAGACCGGATCCTGGGAAACAGGCTGGGATGGTCCCGTAA
GTTCGGGCGGTGCCCTTCGGGGGTAACCCCACCGCCGACGTATCACCTCACACGCTGTTGGCCGATAAGCC
GAGGTATTGGAACCGCACCACTCTGGGGGTTCCCTCTCCCTATGAGGTGGAGGCATAGCACGCCTTAAGTG
TCTACGATCCGGCGTTCACGTGCCGGTGGTCTCCCTAGTTAACGTGGTGCCACAGTCTGTGGTAGGGGCAG
CTGGTCGGATTCCAGCAGAAACCAAGTAGCAAGTGGACGTTGCTACCTCGAGCCTAGAGTCATGGGCCCGC
ATCGACGTTTGCGGTAACTCACAGCGGGCGATAATAGCTGTTGAGAATGTCGGTTCCTGACTTAGCGGGAGA
TGAGCACTCTCTCTCGCGCCC

>SeqID [organism=Tobacco bushy top virus] [moltype=genomic RNA] [isolate=YKMPL] tobacco bushy top virus isolate YKMPL, complete genome

GGGTTACAATATGGAGTTCATCAACAAGATAAAGCAATTGTTGGCAATGAATTTCAAGCCCTCTAAGGGCGT
AATGTCTCGGGAGGAGCTCCGTGAAGCTTTCGATCCCACTTGGGAGCTCCTCATCACACAGGCTCGTGTCAC
CACTGAGGTGTCACGCCAGTGTGAGGATTGGTACACTCTAGCTGTACCCACCACCTACCGCTTGCCAGAGTT
GGCGGTAGAAGAGGCTGTGCGTGAAAAGAACATAGCGCGCGAGGTGGCGGCCAAGTGCCCCCCTGAGGA
CCCGACTCCTGCTTCCCCGCAAGAGCCACAGCCTGTCCCCTTGGCGGAGAGCTCCGAGTGCTCGAGCCAGG
ATGCCGAGCGTGAGGCGCTCGATGAGATATGGGGGCTCCCCACTCCCGCGCCACATCCTCTGCCCAAGTACT
TCGAGCGGCGTTACCAGGCGCTGGCTCGGACCTGCCAGAAGGACTACTCCAACTGGCAGATTGTGCCCTAC
ACGGGTGGGCCACGCGTGCTAAGAGAGGAGGACGTGCTACCAATGGCCACGGGGGTAATACCCTTGCCAT
CACCCCCCCCTAAGGCTTCTGTAATTGGAGCAGTCTTGGGGCTGATTACCCGCCTCACCAAGGTGGCGGGG
GAAGTGAAGGGCAGGCTGAGCGCACCGACGCGGGAGCCCTCGCCCACTTGCTACGGTCTAGAGCAGGTG
GCTGGTGAGCCCATGGGCTACATGAATGCACACTCTGTAGCCATGGAGTTGCGGGCTAGGTACGGAGTTCA
GCCCGCCACAGCTGCGAACTTGCAGCTCGGAAACCGGGTGGCCAGGGAAATCCTGGAAAAACAGTGCGG
GGCCACCCGTGACATGGTGTTCATACTCGGGCACCTCGCCACCACCTTGTGGTTCACCCCCACTATGGTGGA
CTTGGCCCTTCAATGTGGGCCTAAGGATTTTTGCTAGGGGATGTGGTCGCTCGGAGGGGTGTAGAAACTAA
AGTGAAGACGAAAATCCACCCCAAAATCCGAGTGCTTAGGGCGGCCCGTCCCCGGCCCGTAGAAAGAGTGT
CGTACCAAATCGACGTGGTGCGGCCCTGTGCTGACTTTGGAGTCCACAACAACTCCCTCAACAATTTAGTGC
GAGGGGTTAACGAGCGGGTGTTCTACACAGACCACAAGAGGAAAGAGCCCCGCAGACCTTCAGCTGGTA
GTTTTGACAAGATCGACATCAGCGAAATAAAAGCGTTCAGAGTCCAGCCGTGGACTCTCGAGGAGGTCGTT
GACAGCTACACGGGTAGCCAGAGGGTGCGGTATGGACAAGCTGTTGAATCCTTAGCAGTAACGCCCCTCTC
ACGAAACGACGCCCGGGTCAAAACATTTGTGAAGGCGGAAAAGATAAACTTCACCGCCAAGCCCGACCCG
GCTCCTCGTGTTATCCAGCCGCGGGATCCAAGGTTCAATGCCTGCTTTGCCAAATACACAAAGCCCTTGGAA
CCCCTCCTATATAAGCAGCTGGGTAAGCTTTACCAGTTCCCATGCATCGCAAAAGGCTTTAACGCCGTAGAGA
CCGGAGAGATAATAGCCAAGAAGTGGAAGTGCTTCAGTGACCCTGTCTGTGTGGGTTTAGATGCCTCCCGG
TTTGACCAACATGTGTCATGCGATGCACTACGGTTCACCCATAGCGTGTACAAACGGTTCGTGAAGGGCAGG
GAAGTGAACAAGTTGCTTTCCTGGATGTACAAAAACCACGCTCTGGGAAGTGCGAAGGACGGATTCGTCAA

GTATGAGGTGGAAGGCTGTCGGATGAGTGGGGATATGGATACAGCCCTAGGGAATTGTGTCCTGATGGTCC
TCATGACTAGGCAGCTATGCCAGAACCTCTCCATACCGCACGAACTGATGAACAACGGCGACGACTGCATAG
TTATATTTGACAGGCAGTACCTGTCCACCTTTCAGGACGCAGTCGAGCCTTGGTTTAGGGAACTAGGGTTTA
CAATGAAGGTCGAGGAGCCAGTCTACCATCTCGAACGAGTAGACTTTTGCCAGACCCGTCCCGTGTATGACG
GCAAGAAGTGGAGAATGGTCAGGCATATCTCAAGTATAGCCAAGGATTGCTGTTCAGTTATTGACTGGGAAC
AGTTACCGGCTTGGTGGAACGCCATTGGAGAATGTGGCATTGCCGTGGCTGGTGGCATACCCATACACAAC
AGCTTCTTGAGATGGCTCCTGAGATCAGGTGAGAGCAACCCTGATCTCCTGAAGCATGGCGCATGGAAAAA
TGAGGGCCTAGCGTGGTACCGGATGGGCATGGACCTATCCCATGAGAGACACGTTAGTGATGAAGCGCGCG
CCAGCTTCCACACTGCCTTTGGAATCGAACCATCCATGCAGGTCGCATTAGAGCAGATCTATGACTCATTGCC
TGCTCCCACCATTGGTGGGAAACGAGCCAGAGTGTGTAAACCTGGAGAAATGGTATTGGTTGATTCACTCCC
ACCGCGGCACTTTAATAATTACTTCCAGGATGTTGGAATGGGGGGGAGCAGTAGTGATTACGTTGTCCCGG
GGACCCACGAGTTCGAACCGGGGACGTTGTGGACACAATGCTAGTCGATTGAGTTAGCCAGGCATATGGTT
GCAAGCGCTGGGGACTTAGTCCATAAAGCCACTGGTAAAGAGCCTCCAGTAGGCAGGCCCGCCTTGATGA
GTATCAACGAGTGGACACCCGGGCAGCCGCGGACAATGCCCGACGGGTTTCAATGTCTACGATCATAAATGT
CAACACTCATCCACAAGGCAGATACGAAAGAGGAGCTGCTCAACGCTCTATACGGGGAAGTGACGTTAAGG
GAGCTCGAGGAATCAAACCTCGGCGTACTAACCCCCCACCGGGCCGAAAACAAGGTGGTAATGACCCCACT
GCTGCCACCCAGAAGTCAAGGAAGAATCGCCAGCGTCCTGAAACGGTTCCGCCCCACAAAACACACGGGG
GGACTTTTATTCATAGAGAAGATTGTGGTGGTGTTCACGCCCCACATACCGGACGATGCTGGCGGGGAGGT
AGAAATCTGGGTCCACGACAACATGCTACCACACCTAAACAGCGTTGGGCCCCGCGTAAGATTTCCGATGA
GCGGAGGGCCCAGGTTGATAGCATTTTATCCCCCCTACTCGATACCCTTAAGTTGTCAAGTGAGGGGGGCGC
CCCGGAGCTACTTCATTGTATCGGAGTACTCCGGCGTGAACTTCGTCGCAGGCGCGAGCCCGTTCAGCCTGT
ACATAATGTGGGAGCCGAAGATAGAGTGCGTAGCGCACAACTATCTGATGAGGCCCCCCAAAGCGATACCG
ATTTGCAGACATCTGGTGAAGGACAGTCTGTCCTCCCTAACCCTAACTCAGGGGGCACTGAAAAGTGCAAT
GTCGAATCGGTATGCAACAACTGCGACCGGGCTCCCACCACCAACAAGTGGTGAGCAGGATATGGAGGTAG
TGTCCCATCCTCCAGGCTAAATTGGACACAAAACGGAGCTAGATTTAGTGTTGCAATTGCTAGACCGTTAGTC
ACTACGCTAGACAGTGCGACAACTGAGGTCGTCTATGACCCACAGGGTGCCATCCTGTGCAATTTCTAACCC
TTGGCAAGGGTGATCCTAGAGGACGCGGGCATTGAATTGCCTGGGGATGCTTCTAGTTACATACCGGAGTG
GTTGATAGACCTCTGTGTAAGGAAACAGGTGTGAGACCGGATCCTGGGAAACAGGCTGGGTTGGTCCCGTA
AGTTCGGGCGGTGCCCTTCTGGGGTAACCCCACCGCCGACGTATCACCTCACACGCTGTTGACCGACAAGC
CGGGGTATTGGAACCGCACCACTCTGGAGGTTCCCTCTCCTTATGAGGTGGAGGTACAGCACGCCTTAAGT
GTCTGCGACCCGGTGTTCACGTGCCGGTGGTCCCCCTAGTTAACGTGGTGCCACAGTCTGTGGTAGGGGTA
GCTGGTCGGATTCCAGCATAAACCAAGTAGCAAGTGGACGTTGCTACCTCGAGCCTAGAGTCATGGGCCCG
CATCGACGTTTGCGGTAACTCACAGCGGGCGATAATAGCTGTTGAGAATGTCGGTTCCTGACTTAGCGGGA
GATGAGCACTCTCTCTCGCGCCC
>SeqID [organism=Tobacco vein distorting virus] [moltype=genomic RNA] [isolate=YBSh] tobacco vein distorting virus isolate YBSh, complete genome
ACAAAATATAAGAAGGGAGAGACCCTTGCTTGAGTTTTCTTCGAAGCTTATGAACTTTGAATTAATCAACGG
AAGCCATTTGAAAGTTTCCACAACTCGCAAGCTATCTTATAAAGAGAGACTTTTGAACTTAGCTATTTTCCTG
AGTCAATTCGTCAATAAATTCGAGGAAAATGCAACCACAAACACTTTTCTTCGTTCTCTTTGCGCTCTTCTCCC
TCTCCTTCTCAGCAGCAGATGTCCCTTTATTTCAGGGAATGCCCCGCACCGGAACAGCAAGCGGGAACGAA
AGCGGATTTCACGGCTCGCTATTTATAGTGGAGCCAATATCACTCCCTGCTGTGGAGAAGGTACCTCTTCCCC
CACCTGGCTCCAAACTGATAACAAACTCGGAGCCCCACGAGCCCACCTTAAGAGATGCGCTGGTTCTGCTTT
GGCAAGTTACCTCCAGAGATTCCAGACGCCTCTATTCCAAGGCGCAGAACAGTTTAGTAAATTCTTGCGCGT
CTGGACTTGCCATTGCGAAAACGTGGTCGCAAGGAGCCTTCCGAAGCTTCCTTTGGGCCGTGGTCTCAATTT

GGAGCTTGAGCATTTGGGTCATTGTCTCCTGGATCTTCTATCTGGTGACGACATTTACCATGCCCGTCGTATGT
CTCGTATTGCTTTATGCCTTCACAACATTTATGGTGAAGGCGTTGCAGTGGATGTTTACCGGCTGGCCCACTT
GCCTCGCTCTGCTTATCCTAAGAGTGGGGAAGACTATCTTCACGGTTCCCAGATTCAAAAGGAACTACAGTG
AAGAGAAACAGGTTAAGGGATTTGTCTCCTTGAAAATCCCCCAAAGCCCCCCCCGTGGCTGTGTGCTACTCG
TGCAACATGAAGATGGATCTCATGCCGGGTATGCCAGTTGCGTCAAGTTGTTCGACGGCACTTTAGCCCTCA
TGACTTGCCACCACGTGGGCACAGGGGTTCCCAAAGGAAAGGTGGCTTCCTCAAAGACCACTAACAAAATA
CCACTCAGCCTATTCACACCTCTGATCTCTTCCGAAAAAGGAGATTTCATGTTAATGAGCGGACCACCAAACT
GGGAGAGTCTGCTCGGCTGTAAAGGAGCCACCTTTGTTCCCGCATCACAACTTGCTAAATCCAAGATGCGG
TTCTTTTTCATTGAGAAGAACGAATGGATGGCTGATCACGGAGAGATTGTAGGTCCCCGTGATCACTGGTTT
GCTACCACACTCTGTAACTCAGAGCCCGGACACTCCGGCACCCCTATCTTCAATGGTAAATCCATAGTGGGTG
TGCATGCAGGAGGAGAAAATGAGCAGAACTTCAATGTAATGTCAACGATCCCTCCCATCCCCGGTTTGACGA
CCCCGCAGTACGTCTTTGAAACTACTGCTCCTCAAGGTCGGGTCTTTACTGATGAGGACCTGGGTGAGATGC
TGCAAAGCGTTCACTCATCCGTACCCCAGCTGCATAAATTTTTGAGCAAGACTGGGAAGAACTGGGCGGATT
ATTCCGACGAAGAAGATTTTAGTGTGGAAATCAAAAACGAACCAGTCCAGGAGCCCGCGGCAGAAACAAC
GCCGCCTCAACTTGATACTATAGAGGCTCCGCCGGTGTCTGAATCCCGGCAGTGGTACCCTCGTCTTCCTGA
GACGGGAAACGAGAAGGGCAGAGCTGTCTGCCAAAACAACAGCACCCCAGCCTCAGCACAAAACAAGGG
CAAGGAAAAAGAGGTGGTGGCCGAAAAGGCCCCCGCAAACTCCACCCAGCCGAAGCTTCCAGAATCAGCC
GACGGCGGCAAAGAATGTTTAGAGAAGCTTTTGGAGAAAATGGTGGAAAGGATCGACCTTTCTGCTATAGA
AAAGAAAGTGGTGGAAGTATTAGCCCAGAAAGCCATGAAGAAGCCTCGAGGATCCCAGCGGCGCAGACGA
CCGCAGAAAACTTCCGACGATATTTCGAAGGAAAGTACAAATGGGAGGTACCAGCCTCCCCACAAGAGATC
CCCGGCTTTGAAGAATGCGGAAGCCTCCCCCAGTACTACCACCCAAAGCAAATCAAAAGTAGTCAATGGGG
CACCCAACTCATCCAAGAATACCCGGAGCTGGGTGAAAAAGTCTCCGGCTTCGGCTGGCCGTCAGTAGGCC
CCCAAGCTGAGGTGACCTCTCTCACTTTACAAGCAGAGAGGTGGCTGCAACGCGCGCAGTCAGCTAAAATC
CCGTCATCTGAGGACCGGGAGCGCGTTATTAACAAAACAGTGGAGGCTTACTCAAATGTTAAAACTTATGGC
CCTACTGCTACTAGAGGAAATAAGCTCGAGTGGCGGCAATTCATTGAAGATTTTAAGTCAGCCGTCTTTTCTC
TGGAGCTTGATGCAGGTATAGGCGTACCATATATCGCCTATGGAAGGCCCACTCATAAAGGTTGGGTGGAAG
ACCCGAAACTCCTGCCAGTGCTTGCCCGACTCACTTTCAACCGACTACAGAAGATGTTGGAAGTTGAGTCTT
CTGAAATGAGTGCTGAAGAGCTTGTTCAAGCTGGTCTCTGTGATCCTATAAGGACATTTGTAAAGAGAGAAC
CGCACAAGCAATCTAAACTCGATGAAGGCCGCTACCGCCTCATCATGAGTGTTTCCCTAGTAGATCAACTGGT
AGCCCGGGTTCTGTTCCAAAATCAGAACAAGCGAGAAATCGCTCTTTGGAGGGCAAACCCCTCAAAACCCG
GTTTTGGCTTGTCTACGGATGAGCAAGTGCTGGAGTTCGTACAAGCTCTGGCCGCGCAAGTGGAAGTCCCA
CCTGAGGAAGTGATTACCTCCTGGGAGAAGTACCTTGTGCCGACTGACTGCTCTGGTTTCGACTGGAGCGTT
GCGGAATGGATGCTACACGACGATATGGTCGTCCGCAACAAACTCACATTGGACCTGAATCCGACAACGGA
AAAGCTGCGCTTTGCGTGGCTAAAATGCATTAGTAACAGTGTCCTTTGTTTGAGCGATGGCACCCTGCTAGC
CCAAAGAGTCCCCGGTGTTCAGAAATCTGGGAGTTACAACACAAGTAGCTCTAACTCCAGAATCCGGGTTAT
GGCCGCTTATCATTGCGGAGCCGACTGGGCCATGGCCATGGGAGATGATGCCCTCGAGTCAGTCAACACCA
ACCTAGAGGTGTATAAAAGTCTAGGTTTCAAAGTCGAGGTTTCAGGACAACTGGAATTCTGCTCTCACATTT
TTAGAGCGCCTGACCTCGCCCTCCCAGTGAATGAGCGTAAAATGCTGTACAAGCTCATCTTCGGTTACAATCC
TGGGAGCGGGAGTCTGGAGGTGATCTCCAACTATATTGCTGCCTGTGCATCTGTGTTAAACGAGTTGCGGCA
TGACCCAGACTCAGTTGCTCTGCTCACCCAGTGGCTAGTCCATCCAGTGCTGCCACAAAACGATTAAAGGAG
AGAGCACATATAAACTAGCCAAGCATACATCAGTTGCAAGCGTTGGAAGTTCAAGTCTGATTACCAAAGCCC
GACACCATAGATTTTAAATTTTTAGCAGGATTTGCGTCAGGATTTCTATCCGCAATCCCAATTTCAGTTGCGG
GCATTTATTTAGTCTACCTTAAAATCTCAGCCCACGTTAGAGCTATTGTTAATGAATACGGGAGGAGTCAGGA
GTAATAATGGAAATGGTGGATCACGAGTCTCTCGCCCTCGCAGACGCGCACGATCGGTTCGGCCGGTCGTT

GTGGTCGCACCCCCTCGGGGAGCACGGCGAAGAACTCGAAGAAGACGAAATGGAGGCAGGAACAGAAG
AAGCCGTAATGGAGTTGGAGGAAGGTCAAGCAACAGCGAGACTTTCATCTTCAACAAGGACTCAATCAAG
GATAGTTCCTCAGGCTCAATCACTTTCGGGCCGTCTCTATCAGAGAGCGTCGCGCTTTCAGGTGGAGTTCTC
AAAGCCTACCATGAATATAAGATCACAATGGTCAACATACGCTTCATCAGTGAATCCTCTTCCACAGCGGAGG
GCTCCATCGCTTACGAGCTGGACCCCCACTGCAAGCTTTCTAGTCTCCAATCAACCCTCCGTAAATTCCCCGT
CACCAAAGGCGGGCAAGCAACGTTCAGGGCTGCGCAAATTAATGGGGTAGAGTGGCATGATACAACCGAA
GATCAATTTAGGCTGCTCTATAAAGGCAACGGAACAAAAGGTGTTGCCGCCGGGTTCTTTCAAATCCGGTAC
ACCGTGCAATTGCACAACCCCAAATAGGTAGACGCCGAGCCAGGACCTAGCCCAGGACCTCAACCTCAACC
CACACCCTCACCTTCTCCTCAAAAGCATGAGCGCTTCATTGCATACGTCGGCATTCCAATGTTGACAATTCAA
GCGCGGGAGAACGACGACCAGATCTTATTAAGATCCATGGGTTTGCAACGGATGAAGTATATAGAGGACGA
GAATCAAAACTATACGAACATCGATTCGCAGTTTTATTCTCAGTCTAATGTCAATGCCGTCCCCATGTATTATTT
CAACGTGCCAAAAGGTACATGGTCAGTCGATATCAGCTGTGAAGGTTATCAGCCAACAAGCAGCACCACGG
ATCCAAACCGTGGGAGGAGTGATGGGTTAATAGCTTATTCCAATAGTGACTCAGATTACTGGAACGTAGGAG
AAGCGGATGGTGTTAAAATTTCGAATTTGAGGAATGATAACACGTACCGCCAAGGACATCCAGATCTTGAGA
TCAATTCCTGTCATTTTAGAGACGGGCAACTGTTAGAACGTGATGCAACTATAAGTTTCCACGTCGAAGCCCC
AGATGATGGGCGTTTCTTTCTAATAGGACCCGCGATTCAGAAAACCGCCAAGTACAATTATACAATATCCTATG
GCGAGTGGACAGATAGAGATATGGAATTAGGGCTAATTACAGTAGTGCTAGATGAGCACCTTGAGGGTTCT
GGCTCGGGTAATTTGGCGAGGAGAGAAAGGAGATCATTGCATCAACATTGCAAATCTCTCTACGCCCCGGA
AGGACCACCGGAAAACAAACCCTGGGATGACAACCAGATCATTGTTGGAGAAAGACAACTCCGTAAAACTC
TGTCTATGGACATGTCAGAAACTGGTTCTGACCTGAGAGAAGGAAAACTCGAACCAAACCCTAGTGGTGATA
CCAGGGATGAGTTGAATTACACGTTGAGTCTTAGTGACTCCGAAGATGAGCTCATTGACGAGCCAAACTTCG
TTCCTGGAATGCGGATGAATTTAGAGGCAGTACCTTTAGCTAATCCCGCTGACAAAGACTACATACTTCGCGA
CAGAGGCATAGCAACGACTTTGGTCGAACCAGCCTATATGCAAGGTGTAGCTCCTCAGAACAAAATTGAGG
AGGATCCCTGGAAACATGTCCGGGATTTCAAATCTGCTCACACTAGTAAAGGCCCGGCTTCTGTAGCTTCTG
GGTCTACAATGAGAGGTAACTTAAGAGGAGGTACTTTAAGAGGAAGATCTATGCCCCCACCTCTCCCCCCTC
CAGCTGAGAAAGTTGGGGACTCCCTGCCTTCGGGCAAACTTCAATGGGATCCCCCAGGCTCGCCCCTTACT
CCTTTTAAGGATAAGGAACTGGCCAAACCGCCTTCTGATGCTCGTAGCTCGATTTCTGGTCGTTTGACTGGA
GGTCTCCTGAAAAGCCGAGTAGATAAAGATTTGGCGAGTCTGACCACAGCTCAAAGACAGCAGTACGAAAT
AACTAGACAAACTCTTGGTAAGATTGCTGCTAAAGAGTTCCTACAAAGATGTAAGGAGGGTGGTTCATAGTC
TAAGCTCTACGACTATAAAATAACGAGCACACTGAACTCCAAGCCTGTTAGAGTATAACCCAGGCAGTCTGA
ACTCTAAGACTTTAAAACTGTGAGTAATCCT

>SeqID [organism=Tobacco vein distorting virus] [moltype=genomic RNA] [isolate=YKMPL] tobacco veindistorting virus isolate YKMPL, complete genome

ACAAAATATAAGAAGGGAGAGACCCTTGCTTGAGTTTTCTTCGAAGCTTATGAACTTTGAATTAATCAACGG
AAGCCATTTGAAAGTTTCCACAACTCGCAAGCTATCTTACAAAGAGAGACTTTTGAACTTAGCTATTTTCCTG
AGTCAATTTGTCAATAAATTCGAGGAAAATGCAACCACAAACACTTTTCTTCGTTCTCTTTGCGCTCTTCTCCC
TCTCCTTCTCAGCAGCAGATGTCCCTTTATTTCAGGGAATGCCCCGCACCGGAACAGCAAGCGGGAACGAA
AGCGGATTTCACGGCTCGCTATTTATAGTGGAGCCAATATCACTCCCTGCTGTGGAGAAGGTGTCTCTTCCCC
CACCTGGCTCCAAACTGATAACAAACTCGGAGCCCCACGAGCCCACCTTAAGAGATGCGCTGGTTCTGCTTT
GGCAAGTTACCTCCAGAGATTCCAGACGCCTCTATTCCAAGGCGCAGAACAGTTTAGTAAGTTCTTGCGCGT
CTGGACTTGCCATTGCGAAAACGTGGTCGCAAGGAGCCTTCCGAAGCTTCCTTTGGGCCGTGGTCTCAATTT
GGAGCTTGAGCATTTGGGTCATTGTCTCCTGGATCTTCTATCTGGTGACGACATTTACCATGCCCGTCGTATGT
CTCGTATTGCTTTATGCCTTCACAACATTTATGGTGAAGGCGTTGCAGTGGATGTTTACCGGCTGGCCCACTT
GCCTCGCTCTGCTTATTTTAAGAGTGGGGAAGACTATCTTCACGGTTCCCAGATTCAAAAGGAACTACAGTG

AAGAGAAACAGGTTAAGGGATTTGTCTCCTTGAAAATCCCCCAAAGCCCCCCCCGTGGTTGTGTGCTACTCG
TGCAACATGAAGATGGATCTCATGCCGGGTATGCCAGTTGCGTCAAGTTGTTCGACGGCACTTTAGCCCTCA
TGACTTGCCACCACGTGGGCACAGGGGTTCCCAAAGGAAAGGTGGCTTCCTCAAAGACCACTAACAAAATA
CCACTCAGCCTATTCACACCTCTGATCTCTTCCGAAAAAGGAGATTTCATGTTAATGAGCGGACCACCAAACT
GGGAGAGTCTGCTTGGCTGTAAAGGAGCCACCTTTGTTCCCGCATCACAACTAGCTAAATCCAAGATGCGGT
TCTTTTTCATTGAGAAGAACGAATGGATGGCTGATCATGGAGAGATTGTAGGTCCCCGTGATCACTGGTTTG
CTACCACACTCTGTAACTCAGAGCCCGGACACTCCGGCACCCCTATCTTTAATGGTAAATCCATAGTGGGTGT
GCATGCAGGAGGAGAAAATGAGCAAAACTTCAATGTAATGTCAACGATCCCTCCCATCCCCGGTTTGACGAC
CCCGCAGTATGTCTTTGAAACTACTGCTCCTCAAGGTCGGGTCTTTACTGATGAGGACCTGGGTGAGATGCT
GCAAAGCGTTCACTCATCCGTACCCCAGCTGCATAAATTTTTGAGCAAGACTGGGAAGAATTGGGCGGATTA
TTCCGACGAAGAAGATTTTGGTGTGGAAATCAAAAACGAACCAGTCCAGGAGCCCGCGGCAGAAACAACG
CCGCCTCAACTTGATACTATAGAGGCTCCGCCGGTGTCTGAATCCCGGCAGTGGTACCCTCGTCTTCCTGAGA
CGGGAAACGAGAAGGGCAGAGCTGTCTGCCAAAACAACAGCACTCCAGCCTCAGCACAAAACAAGGGCA
AGGAAAAAGAGGTGGTGGCCGAAAAGGCCCCCGCAAACTCCACCCAGCCGAAGCTTCCAGAATCAGCCGA
CGGCGGCAAAGAATGTTTAGAGAAGCTTTTGGAGAAAATGGTGGAAAGGATCGACCTTTCTGCTATAGAAA
AGAAAGTGGTGGAAGTATTAGCCCAGAAAGCCATGAAGAAGCCTCGAGGATCCCAGCGGCGCAGACGACC
GCAGAAAACTTCCGACGATATTTCGAAGGAAAGTACAAATGGGAGGTACCAGCCTCCTCACAAGAGATCCC
CGGCTTTGAAGAATGCGGAAGCCTCCCCCAGTACTACCACCCAAAGCAAATCAAAAGTAGTCAATGGGGCA
CCCAACTCATCCAAGAATACCCGGAGCTGGGTGAAAAAGTCTCCGGCTTCGGCTGGCCATCAGTAGGCCCC
CAAGCTGAGGTGACCTCTCTCACTTTACAAGCAGAGAGGTGGCTGCAACGCGCGCAGTCAGCTAAAATCCC
GTCATCTGAGGACCGGGAGCGCGTTATCAACAAAACAGTGGAGGCCTACTCAAATGTGAAAACTTATGGCC
CTACTGCTACTAGAGGGAACAAGCTCGAGTGGCGGCAATTCATTGAAGATTTTAAGTCAGCCGTCTTCTCTC
TGGAGCTTGATGCAGGTATAGGTGTACCATATATCGCCTATGGAAGGCCCACTCATAAAGGTTGGGTGGAAG
ACCCGAAACTCCTGCCGGTGCTTGCCCGACTCACTTTCAACCGACTACAGAAGATGTTGGAAGTTGAGTCTT
CTGAAATGAGTGCTGAAGAGCTTGTTCAAGCTGGTCTCTGTGATCCTATAAGGACATTTGTAAAGAGAGAAC
CGCACAAGCAATCTAAACTCGATGAAGGCCGCTACCGCCTCATCATGAGTGTTTCCCTAGTAGATCAACTGGT
AGCCCGGGTTCTGTTCCAAAATCAGAACAAGCGAGAAATCGCTCTTTGGAGGGCAAACCCCTCAAAACCCG
GTTTTGGCTTGTCTACGGATGAGCAAGTGCTGGAGTTCGTACAAGCTCTGGCCGCGCAAGTGGAAGTCCCA
CCTGAGGAAGTGATTACCTCCTGGGAGAAGTACCTTGTGCCGACTGACTGCTCTGGTTTCGACTGGAGCGTT
GCGGAATGGATGCTACACGACGATATGGTCGTCCGCAACAAACTCACATTGGACCTGAATCCGACAACGGA
AAAGCTGCGCTTTGCGTGGCTAAAATGCATTAGTAACAGTGTCCTTTGTTTGAGCGATGGCACCCTGCTAGC
CCAAAGAGTCCCCGGTGTTCAGAAATCTGGGAGTTACAACACAAGTAGCTCTAACTCCAGAATCCGGGTTAT
GGCCGCTTATCATTGCGGAGCCGACTGGGCCATGGCCATGGGAGATGATGCCCTCGAGTCAGTCAACACCA
ACCTAGAGGTGTATAAAAGTCTAGGTTTCAAAGTCGAGGTTTCAGGACAACTGGAATTCTGCTCTCACATTT
TTAGAGCGCCTGACCTCGCCCTCCCAGTGAATGAGCGTAAAATGCTGTACAAGCTCATCTTCGGTTACAATCC
TGGGAGCGGGAGTCTGGAGGTGATCTCCAACTATATTGCTGCCTGTGCATCTGTGTTAAACGAGTTGCGGCA
TGACCCAGACTCAGTTGCTCTGCTCACCCAGTGGCTAGTCCATCCAGTGCTGCCACAAAACGATTAAAGGAG
AGAGCACATATAAACTAGCCAAGCATACATCAGTTGCAAGCGTTGGAAGTTCAAGTCTGATTACCAAAGCCC
GACACCATAGATTTTAAATTTTTAGCAGGATTTGCGTCAGGATTTCTATCCGCAATCCCAATTTCAGTTGCGG
GCATTTATTTAGTCTACCTTAAAATCTCAGCCCACGTTAGAGCTATTGTTAATGAATACGGGAGGAGTCAGGA
GTAATAATGGAAATGGTGGATCACGAGTCTCTCGCCCTCGCAGACGCGCACGATCGGTTCGGCCGGTCGTT
GTGGTCGCACCCCCTCGGGGAGCACGGCGAAGAACTCGAAGAAGACGAAATGGAGGCAGGAACAGAAG
AAGCCGTAATGGAGTTGGAGGAAGGTCAAGCAACAGCGAGACTTTCATCTTCAACAAGGACTCAATCAAG
GATAGTTCCTCAGGCTCAATCACTTTCGGGCCGTCTCTATCAGAGAGCGTCGCGCTTTCAGGTGGAGTTCTC

AAAGCCTACCATGAATATAAGATCACAATGGTCAACATACGCTTCATCAGTGAATCCTCTTCCACAGCGGAGG
GCTCCATCGCTTACGAGCTGGACCCCCACTGCAAGCTTTCTAGTCTCCAATCAACCCTCCGTAAATTCCCCGT
CACCAAAGGCGGGCAAGCAACGTTCAGGGCTGCGCAAATTAATGGGGTAGAGTGGCATGATACAACCGAA
GATCAATTTAGGCTGCTCTATAAAGGCAACGGAACAAAAGGTGTTGCCGCCGGGTTCTTTCAAATCCGGTAC
ACCGTGCAATTGCACAACCCCAAATAGGTAGACGCCGAGCCAGGACCTAGCCCAGGACCTCAACCTCAACC
CACACCCTCACCTTCTCCTCAAAAGCATGAGCGCTTCATTGCATACGTCGGCATTCCAATGTTGACAATTCAA
GCGCGGGAGAACGACGACCAGATCTTATTAAGATCCATGGGTTTGCAACGGATGAAGTATATAGAGGACGA
GAATCAAAACTATACGAACATCGATTCGCAGTTTTATTCTCAGTCTAATGTCAATGCCGTCCCCATGTATTATTT
CAACGTGCCAAAAGGTACATGGTCAGTCGATATCAGCTGTGAAGGTTATCAGCCAACAAGCAGCACCACGG
ATCCAAACCGTGGGAGGAGTGATGGGTTAATAGCTTATTCCAATAGTGACTCAGATTACTGGAACGTAGGAG
AAGCGGATGGTGTTAAAATTTCGAATTTGAGGAATGATAACACGTACCGCCAAGGACATCCAGATCTTGAGA
TCAATTCCTGTCATTTTAGAGACGGGCAACTGTTAGAGCGTGATGCAACTATAAGTTTCCACGTCGAAGCCCC
AGATGATGGGCGTTTCTTTCTAATAGGACCCGCGATTCAGAAAACCGCCAAGTACAATTATACAATATCCTATG
GCGAGTGGACAGATAGAGATATGGAATTAGGGCTAATTACAGTAGTGCTAGATGAGCACCTTGAGGGTTCT
GGCTCGGGTAATTTGGCGAGGAGAGAAAGGAGATCATTGCATCAACATTGCAAATCTCTCTACGCCCCGGA
AGGACCACCGGAAAACAAACCCTGGGATGACAACCAGATCATTGTTGGAGAAAGACAACTCCGTAAAACTC
TGTCTATGGACATGTCAGAAACTGGTTCTGACCTGAGAGAAGGAAAACTCGAACCAAACCCTAGTGGTGATA
CCAGGGATGAGTTGAATTACACGTTGAGTCTTAGTGACTCCGAAGATGAGCTCATTGACGAGCCAAACTTCG
TTCCTGGAATGCGGATGAATTTAGAGGCAGTACCTTTAGCTAATCCCGCTGACAAAGACTACATACTTCGCGA
CAGAGGCATAGCAACGACTTTGGTCGAACCAGCCTATATGCAAGGTGTAGCTCCTCAGAACAAAATTGAGG
AGGATCCCTGGAAACATGTCCGGGATTTCAAATCTGCTCACACTAGTAAAGGCCCGGCTTCTGTAGCTTCTG
GGTCTACAATGAGAGGTAACTTAAGAGGAGGTACTTTAAGAGGAAGATCTATGCCCCCACCTCTCCCCCCTC
CAGCTGAGAAAGTTGGGGACTCCCTGCCTTCGGGCAAACTTCAATGGGATCCCCCAGGCTCGCCCCTTACT
CCTTTTAAGGATAAGGAACTGGCCAAACCGCCTTCTGATGCTCGTAGCTCGATTTCTGGTCGTTTGACTGGA
GGTCTCCTGAAAAGCCGAGTAGATAAAGATTTGGCGAGTCTGACCACAGCTCAAAGACAGCAGTACGAAAT
AACTAGACAAACTCTTGGTAAGATTGCTGCTAAAGAGTTCCTACAAAGATGTAAGGAGGGTGGTTCATAGTC
TAAGCTCTACGACTATAAAATAACGAGCACACTGAACTCCAAGCCTGTTAGAGTATAACCCAGGCAGTCTGA
ACTCTAAGACTTTAAAACTGTGAGTAATCCT
>SeqID [organism=Tobacco bushy top virus satellite RNA] [moltype=genomic RNA] [isolate=YBSh] tobacco bushy top virus satellite RNA isolate YBSh, complete genome
GGGTATCGATACAAGGAGCAGCATGCTCATTCCTCTTAGTTGGAGACTGTGCGGAGGAGTATGCATCTTTTT
GGACGGAGAGATGTTGGCCGTGGGCAGCAACCCGTGGATAGCTGATAAGATCGTCCAGTGGATGGGGACC
TAAGGACCCCCATTGGCTCTCTCTCTTCTGTCAGAGCGGCTTGGTGAGCCGCCAGAGTGAACCGCTCGAGC
TGAATTAAAACCCCTAAGCGGTTCGCTCTGGAGCCTGTAGGGTCCACGTCCATCGCCCTTAGTTGGAGACTG
TGGGTGCTTGGCGTAGCCCTGTGGGTGGCACGCCTTGAAGCTCCGATACCTATCCTATTAGCCCTGGCCGTT
GATACTTGTGGATCCACGAGGAACCACTCGAACGAATCGGATCACCAGGGAGTACCCTTAGGGATGGACCA
GCAACCAACTGGGCATCAACGGGATCGGCTAGACGTAGGACAGCAGTAGGCGGGAGACACTAACGCCGC
GCCTCCTACGGCTCTGCGTAAGCAGACAAGTGAGACTCACCTCAGTCACCGAGTACCTGGATTAAGGGGAA
AGGAGGGAGTCGAATACCGCGTCCCCGGCTGGCGTAACGCGAGCCCCGCGGTAGGAGTAATAGTGCAGCG
GTTAGGAAAGCTGTGCGGCGCCGACGCGACGGAGCCAAATACGGAGGACTGTGGCTAGGCTGCCTTTGGC
CGATGCCGTCATGCACTGGGGTAGTACCCCAGCCCAGAACACCCTGGGGAGTCTCCCTCCACTCGCGGTGC
CCTGAAACGGGCACTACTACCTAATAAGTAGATCCCATCTAGCCC
>SeqID [organism=Tobacco bushy top virus satellite RNA] [moltype=genomic RNA] [isolate=YKMPL] tobacco bushy top virus satellite RNA isolate YKMPL, complete genome

GGGTTTCGATACAAGGAGCAGCATGCTCACTCTTCTTAGTTGGAGACTGTGCGGAGGAGTATGCAACTTTTT
GGACGGAGAGATGTTGGCCGTGGGCAGCAACCCGTGGATCGCTGATAAGATCGTCTAGTGGATGGGGACC
TAAAGACCCCCACTGGCTCTCTCTCTTCTGTCAGAGCGGCTTGGTAGGCCGCCAGAGTGGACCGCTCGAGC
GTAATGAAGACCTATAAGCGGTCCGCTCTGGAGCCTATAGGGTCCATGTCCTTCACCCTTAGTTGGAGACTGT
GGGTGCTTGGTGTGGTCCTGTGGGTGGCCCGCCATGAAGCTCCGAAACTCAACCCTTTAGCCCTGTCCGTT
GATACTTGTGGACCTCGCAGGTACCACTCGAACGAATCGGATACACAGGGAGTGCCTAGGGGATGGACCAG
CTTCGAACTGGGCATCAACGGCATCGGCTAGACACAGGACAGCAACCGGCGGAAGATACTAACGCCTCGCC
TCCTGTGGCTCTGTGAAAGCAGACACTGTTCGACCCACCTCAGTCACCGAGTACCTGGATTAAGGGGAAAG
GAGGGGGTTGATTGCCGCGTCCCCGGCTGGCGTAAAGCGAGCCCCGCGGTAGGAGTAATAGTGCAGTGGT
TAGGAAAGCTGCGCGGCGCCGACGCGACGGAGCCAAATACGAAGGACTGTGGCTAGGCTGCCTTTGGCCG
ATGCCGTCATGCACTGGGGTAGTACCCCAGCCCAGAACACTCTGGGGAGTCTCCCTTCACTCGCGGTGCCCT
GAAACGGGCACTACTACCTAATAAGTAGATCCCATCTAGCCC
>SeqID [organism=Tobacco vein distorting virus associated RNA] [moltype=genomic RNA] [isolate=YBSh] tobacco vein distorting virus associated RNA isolate YBSh, complete genome
GGGGATTCATGGAGACAGCTAGAGGTTTACTAGACGCCCTAGGCGATCCATGCACCAACGTGGTTCGGGCT
TTCAAGGCACGACCTGTCATATATAGTTTTGACCAGGTCGTACATGCCTTCTCAGCTGAAAACCCCTTCTCAG
GTTGTAGTGAGAGGGTGGTATTAGCTGTACCCCATATCATTATGGGGAATAGTCCATTAGAGCGGCTATTGGA
GCTGCTCGCCACATCTAGGATAGCGGTTGCCCAGTTTAAACGGTCTATAGTAGACTGGGCCCACCGTAATAGT
ATATATCTAGGATTGTCGGCTGCAACTAGTGCAGCCGCCTTAGTAATAGTAGAGAGATGGAGGCTTTCACAA
GCCCCCCCCATGCAAGAGGAACCAGTGCGGTTGGAGGACTGCCTGGAGATTGATGATCCTGACCTCCCCCT
CCTGGAACAAACTGTGCCAGATGAGCCAACCACAGACAGAGCTAGAGCTCAGCTGGCTATCAGAAAGAGG
GTTACCCGTAGAGTGAGGCCCAACTCAACTGCTAGATTCATAAGGGTCCTCAGAGCCGAGGTGAAAGCGAC
AGTCGGAACACCAACGTACACAGCTGCGAATGTGGCTGTAATCCGGCACATTGTCGAGAAGTTCGCTAAGG
AGTACAACATCAGAACATCCTCGTACTCCCACCTCATGGGCGATGTGGTAAGCGCAGTCATGACTCCTTACAA
ATCGGAGTACAGGCAGGCTGCGTACTGCGGGAGTCTCACGAGCCGCCTACGGCGGTGGCTCGTTGAGCTT
AAATAGGGGGGCCTACAGCTGGTGGGTGGGTACGAGCATGATATGCCAGATCTTACAAACGTACCCGGCCT
GGACCCACGACAACTGAGGCCGTCTATAGTAGAGTATAACAAGGAATTCCGTCGTATAGTGGCACCTAGCTA
CAACTACGGAGTGACTTACTTTAATAATAGTAGAGCTAACCTACTGAGGGGTCTTGTCAACAGGGTGTTGAC
ATATAAGGGTGGACCTATCCTCGAGCCTAGTCCGGGCTCCTGGAAGTCGCTTTCCCCGCTCGCAAGGAAGAT
AGGTATTAGGTGCAGAGCCTCCCCGATGAGCCCGGATGAATTTCTGGCGTGCTATGTGGGACGTAAACGCA
CTATCTATACCAAGGCCCTTCGCAGCTTGGAGGACACCCCCCTCAACAATAAAGATTTTGTGGTTCGGGCCTT
CTTAAAGAAGGAGAAAGATAAACTGCTGAGTAGCAGCTCCGATCCCAGAATAATTCAGCCTCGACATCCGAG
GTTTTTAGTTAGTTTTGGTAGATACATCAGACCTCTGGAACAGAAATGCTATAAGGCATTCTCATCGTTGTTCC
AGCGATTTGCCGTGTCTCGGACCCCTGTTTGCTTTAAAGGTCTTAATTACTTGCGCAGGGGCGAGATGCTCTT
AAAGAAGTGGGAAAGCTTCGTTAACCCCGTGGCGATTTGCTTAGACGCATCTCGTTTTGACCTCCACGTGTC
GGTTGAAGCACTCCAGTACACCCACACATTATATTACACGGCATTCGGTAGAGATCCCTTGCTCATGCAACTA
TTGGAGAACCGCTTGGAAACCCGCGGAGTAGCTATTTGCAAGGAAGGATCCTACAGGTACAGGAAGAAAG
GTGGGCGTTGCTCAGGGGATAATGACACATCTTTAGGCAACGTGATTATAATGCTATCTATAACCTACCATCTT
CTTGAGGGACTGCCTTTTCCACACATTGAAGTCGCTAACGACGGAGATGATCAAGTGCTCATTGTGGAAAAA
GAGAATGAGTTAGCCCTGCTGGAGGCAGTTGAGCCCTGCTTCGCGAAATTTGGGTTCCGGCTAACCGTCGA
ACAGCCAGTCTATGAATTTGAGCGCATAGACTTCTGCCAGACTCGGCCTGTTCGATTGAGTCCCGATGTGAC
AACTATGGTCCGACATCCACGCCTCGCCATGACGAAAGATTTGACCTCCTTCATCCCAATAGAACGAGGGTT
GATGAAGTACCACATGCTAAGCGCAATAGGCGCATGTGGTAAGGCTTGTTATAATGATATACCCGTGCTCGGA
GCAATGTACAGAAAGCTTGAGAGCGTGGGACGAGCAGCCGGAGGCAGAGAAGAAAAATGGTGGGCGTCA

ACGAATGCAAACCCCGACTTCCAGAGATTATCAAGTAAAGCAGCGACCAACGTAGGACTTACTGTTGAAGCT
CGCGTATCGTTTTGGAAGGCATTCGATATTCTCCCCGATCACCAAACCGCTCTAGAAAGAGAATGGGAGAAT
TGGAAACCGGACTTTGAGTTGGCTACCGTTCCTGTTTTCGTAGGAACCTATGCTGTTGTAGATGGTGATGTTT
AAAATTAGACCAGTCGCCCCCATCCTCCGCTGGATGGGGGGGAAGACCCGTGGCCGCGGTGTTATGCTAGG
AACTCCCACGTAAGTGGATCGGCCCAAACCATCGAAGTAGCATAGTTATTAAAATATATGTTTAGTCTAGTTTA
GGATCGAGTCTTAAGGCAGCCCCTTGCCAGGTTGCGACTAGGGGGCCATCAGGCCTGGCACACCCGTTGG
TCAGCTCGTAACTACTCTCGTTGGATCGAGAGGACAATCCGAGTAGAGGCAGAGAAGGGCCTCCGGTTTGA
CCATACCGTAATAGCCGGACGCATAGCTATTCGGGCTGGGGACGTCATACCCAGTGCGATAGGTCCCGCAGT
TGGACTAGGACACTTGTCCCGGCCGTTTCTCCAAGTGGCGCGCTTCAATCAGCAAACTCTTCCGCTAGAGTA
TTGCTTAGTAGTAGGCTCTTAGCCAACCTCTTTCAATGTAAGAGGACAGAAGCGTTTTGGTCGGAGGAGAG
ACCGTCGCACCTGAACTCCATCACTGGGAAACTCCAGTGAGGGCGATAGGAGATTCAGGAGGTTTGGAAA
CACTTTGTTTACCAGGTACACCGCCCC
>SeqID [organism=Tobacco vein distorting virus associated RNA] [moltype=genomic RNA] [isolate=YKMPL] tobacco veindistorting virus associated RNA isolate YKMPL, complete genome
GGGGATTCATGGAGACAGCTAGAGGTTTACTAGACGCCCTAGGCGATCCATGCACCAACGTGGTTCGGGCT
TTCAAGGCACGACCTGTCATATACAGCTTTGACCAGGTCGTACATGCCTTCTCAGCTGAAAACCCCTTCTCAG
GTTGTAGTGAGAGGGTGGTGTTAGCTGTACCCCATGTCATTATGGGGAATAGTCCATTAGAGCGGCTATTGG
AGCTGCTCGCCACATCTAGGATAGCGGTGGCTCAGTTTAAACGGTCTATAGTAAACTGGGTTCACTGTAATAG
TATATATTTAGGATTGTCGGCTGCAACTAGTGCAGCCGCCCTAGTAATAGTAGAGAGATGGAGGCTTTCCCAA
GCCCCCCCCATGCAAGAGGAGCCAGTGCGATTGGAGGATTGCCTGGAGATAGATGATCCTGACCTCCCCCT
CCTGGAACAAACTGTGCCAGATGAGCCAACCATTGACAGAGCTAGAGCTCAGCTGGCTATCCGAAAGAGG
GTTACCCGTAGAGTGAGGCCCAACTCAACAGCTAGATTCATAAGGGTCCTCAGAGCCGAGGTGAAAGCGAC
TGTCGGAACACCAACATACACAGCTGCGAACGTGGCTGTAATCCGGCACATTGTCGAGAAATTCGCGAAGG
AGTACAACATTAGAACATCCTCGTACTCCCACCTCATGGGCGATGTGATAAGCGCAGTCATGACTCCTTACAA
ATCGGAGTACAGGCAAGCTGCGTACTGCGGGAGTCTCACGAGTCGCCTACGGCGGTGGCTCGTTGAGCTTA
AATAGGGGGGCCTACAGCTGGTGGGTGGGTACGAGCATGATATGCCAGATCTTACAAACGTACCCGGCCTG
GACCCACGACAACTGAGGCCGTCTATAGTAGAGTATAACAAGGAATTCCGTCGTATAGTGGCACCTAGCTAC
AACTACGGAGTGACTTATTTTAATAATAGTAGAGCTAACCTACTGAGGGGTCTTGTCAACAGGGTGTTGACAT
ATAAGGGTGGACCTATCCTCGAGCCTAGTCCGGGCTCCTGGAAGTCACTTTCCCCGCTCGCAAGGAAGATAG
GTATTAGGTGCAGAGCCTCCCCGATGAGCCCGGATGAATTTCTGGCGTGCTATGTGGGACGTAAACGCACTA
TCTATACCAAGGCCCTTCGCAGCTTGGAGGACACCCCCCTCAACAATAAAGATTTTGTGGTTCGGGCCTTCTT
AAAGAAGGAGAAAGATAAACTGCTGAGTAGCAGCTCCGATCCCAGAATAATTCAGCCTCGACATCCGAGGT
TTTTAGTTAGTTTTGGTAGATACATCAGACCTCTGGAACAGAAATGCTATAAGGCATTCTCATCGTTGTTCCAG
CGATTTGCCGTGTCTCGGACCCCTGTTTGCTTTAAAGGTCTCAATTACTTGCGCAGGGGCGAGATGCTCTTA
AAGAAGTGGGAAAGCTTCGTTAACCCCGTGGCGATTTGCTTAGACGCATCTCGTTTTGACCTCCACGTGTCG
GTTGAAGCACTCCAGTACACCCACATATTGTATTACACGGCATTCGGTAGGGATCCCTTGCTCATGCAACTATT
GGAGAACCGCTTGGAAACCCGCGGAGTAGCTATTTGCAAGGAAGGATCCTACAGGTACAGGAAGAAAGGT
GGGCGTTGCTCAGGGGATAATGACACATCTTTAGGCAACGTGATTATAATGCTATCTATAACCTACCATCTTCT
TGAGGGACTGCCTTTTCCACACATTGAAGTCGCTAACGACGGAGATGATCAAGTGCTCATTGTGGAAAAAG
AGAATGAGTTAGCCCTGCTGGAGGCAGTTGAGCCCTGCTTCGCGAAATTTGGGTTCCGGCTAACCGTCGAA
CAGCCAGTCTATGAATTTGAGCGCATAGACTTCTGCCAGACTCGGCCTGTTCGATTGAGTCCCGATGTGACA
ACTATGGTCCGACATCCACGCCTCGCCATGACGAAAGATTTAACCTCCTTCATCCCAATAGAACGAGGGTTGA
TGAAGTACCACATGCTAAGCGCAATAGGCGCATGTGGTAAGGCTTGTTATAATGATATACCCGTGCTCGGAGC
AATGTACAGAAAGCTTGAGAGCGTGGGACGAGCAGCCGGAGGCAGAGAAGAAAAATGGTGGGCGTCAAC

GAATGCAAACCCCGACTTTCAAAGATTATCAAGCAAAGCAGCGACCAACGTAGGACTTACTGTTGAAGCTC
GCGTATCGTTTTGGAAGGCATTCGACATTCTCCCCGATCACCAAACCGCTCTAGAAAGAGAATGGGAGAATT
GGAAACCGGACTTTGAGTTGGCTACCGTTCCTGTTTTTGTAGGAACCTATGCTGTAGTAGATGGTGATGTTTA
AAATTAGACCAGTCGCCCCCGTCCTCCGCTGGATGGGGGGGAAGACCCGTGGCCGCGGTGTTATGCTAGGA
ACTCCCACGTAAGTGGATCGGCCCAAACCATCGAAGTAGCATAGTTATTATAATGTATATTTAGTCTAGTTTAG
GATCGAGTCTTAAGGCAGCCCCTTGCCAGGTTGCGACTAGGGGGCCATCAGGCCTGGCACACCCGTTGGTT
AGCTCGTAACTACTCTCGTTGGATCGAGAGGACAATCCGAGTAGAGGCAGAGAAGGGCCTCCGGTTTGACC
ATACCGTAATAGCCGGACGCATAGCTATTCGGGCTGGGGACGTCATACCCAGTGCGATAGGTCCCGCAGTTG
GACTAGGACACTTGTCCCGGCCGTTTCTCTAAGTGGCGCGCTTCAATCAGCAAACTCTTCCGCTAGAGTATT
GCTTAGTACTAGGCTCTTAGCCAACCTCTTTCAATGTAAGAGGACAGAAGCGTTTTGGTCGGAGGAGAGAC
CGTCGCACCTGAACTCCATCACTGGGAAACTCCAGTGAGGGCGATAGGAGATTCAGGAGGTTTGGAAACA
CTTTGTTTACCAGGTACACCGCCCC

>SeqID [organism=Tobacco polerovirus virus 1] [moltype=genomic RNA] [isolate=YBSh] Tobacco polerovirus virus 1 isolate YBSh

GAGGAATTGCAGCTCTAAGCGCAACTCTTTACAATCATTGCAATCGTTATAGCACATGTTTGTATTGACTCCAA
CAGGATCAATCGCATTTGACGAAACCAAGAAGCTAACAAAGCTAGTTTTTGTCAATCTAGTGATTGGATTCCC
TACCCTCCTAGCTCAAGCTCACATTCTTTTTCACTACAATTATGAACAAATTTATAACATTTCTCGTAGTGTTTT
CTATCTACTCCCTCTGCTCAGCAGATGCAAAGGCTGGGTTTCATCATCCGGCTTATCCATATCGAGGCACTGG
CATAGCCAATGTCTCAAATGGGGGTTACTATGCGGCACCCATCCCGCTATACAAATTGGGCACACTTCCCTCA
CCATCAAAATTGGTGAGCCCTCCTCCGCTGCAGCTTACAGACGCGAGCTATCAAGAATTGATTCAAGCTCTTA
CGTCCAAAATGCGCGCGGACTCTACAACTCTTGGGGACAAGACATGGCAACATTTGTCAAAAATGCTGTGT
GTTTCCTTGAAATCCGCAAAAGATGCTACCCGAAGCGCGGTCTCAACGCTACTCTGGACTATAATTTCAATTT
GGTTCGGAGTTTATTGGACGCTTGCCAAGATACTGGCCATCTTCTTGTGGACGTTCAGTATACAGTCCTTTTG
CATAATCTTGCTGTGTTTTACAACCAGCTTGATTTTCAAGGCTGTGAAATTCTGTTTCGAGAGCTTACCGGTTT
ACCTGTTTACGTACCCGGCGAAGCTTATTTGGAGGGCAGCTTTCTCCAAAAAGAATTACAAGGATGAGAAA
GCCGTAGAGGGTTTTAGAAGTTTCGTGGTCCCTCAAAAACCACCAAAGTCTGCCGTGATTGAATTACAACAT
GAAAACGGCAGCCACCTGGGTTATGCTAATTGCATAAGATTATATAGTGGAGAGAACGCGCTTGTAACAGCA
GAACACTGCTTGCAGGGTGCGTACGCAGCATCGATGAAAACTGGCAATAGGATTCCGATGTCTAGCTTCATT
CCCATTTACAAAAGCAACCCACGTGACATCGCCATCATGGCAGGACCCCCAAATTGGGAAGGGCTTCTTGCT
GTCAAAGGCGCCAGTTTCCAAACTGCTGACAAAATCGGCAGAGGACCTGCTTCTTTCTTCACGCTAGAGAA
AGGAGAGTGGATGTGCAACAATGCCCAAATAGATGGATCACATGACAAATTTGTGACTGTCTTGTGTAACAC
TGAAGCAGGGTGCTCAGGGACAGGGTTTTGGTCTTCCAAAACTTTGTTGGGCGTGCTCAAAGGATACCCAC
TAGAGGAAGGGTGCAATTACAACGTTATTTCTGTAATACCTTCCATTCCAGGTCTGACCTCCCCTAACTACGT
GTTTGAATCGACTACCATAAGAGGTAGAGTCTTCAGTTCAGAAGCAATCGAAGAGATGGAGAGGGAGGCTA
GGGAGGCAGTGAGAAAGTTGCTGTCCTTCAAGTCTCAAACTGGAAAGAACTGGGCCGATTACTCCGATGAT
GAGGAGTATGGTGATGAAAAGAAGGCAGCTGAAGCGCCAAAGAAGGAAGCTCAAGCGCCCAAGGAGGA
AACTAAAGAGGTTCCCGCAGAGAAAACTGCGCAAACAAACCCACAGGCTCCTTTAAATGGGGTCGCGGCA
CCGCCCGCCATAACAACCGGAACCTCCGCCACCCCAAGCGGCGCTACCAGCGCACCACCGATGGACAAGAT
GGTCGAGCAGATCATCACAGCTATGGTGGGGAGGATAAACCTCTCGGAAATCGAGAGGAAAATAGTGGAA
AGAGTTTCAGAGAAAGCTCTCAAGAAGCCAACTCAAAGCAAGCGAGGAAGGCGTGGAGGGAAGAACAA
GCCAGACAATTCATCTCCTACTTCCGAGCCATCTACACGTGGGGCGCCCAAGAGGGTGCAGCCCCCCCAGG
CTTCAAAAGATGCGGTAGGACCCCGCGTTACTACCACCCCCGTACCAGAGGTGAAACCCAATGGGGGCGCA
AACTCTGCCAAGTTCATCCCGAGCTGGCGAATAAAACAGCAGGCTTCGGCTGGCCAAAGACCGGAGCACA
AGCAGAACTTCAAAGCTTGAATCTGCAGGCTGCTAGGTGGCTCGAACGTGCCGAGTCGTCCACTATACCTAG

TGCAGAAGCGAGAGAGCACGTGATTCAAAAGACTGTGAGAGCGTACCAAAACTGTACAACTCAAGCTCCA
CAGTGCTCCCTTAAATCCAAACTGGACTGGACTGGCTTCCAAAATGATATAAAAGAAGCAGTCCGGTCTCTT
GAATTAGATGCGGGTGTAGGCATCCCTTACATAGCATATGGGCTCCCCACTCACCGAGGATGGGTTGAAGAT
CCCAAGCTCCTGCCGATAATTGCTCAGTTGACCTTCGACCGACTACAGAAGATGTCGGAGGCCAACTTCGAG
TCCATGACTCCGGAGGAGCTCGTTCAAGAGGGGCTTTGCGATCCTATAAGATTATTTGTCAAGGGTGAGCCC
CATAAGCAGAGCAAACTGGATGAAGGCCGCTACCGCCTCATTATGTCAGTTTCTTTAATAGATCAACTGGTAG
CCCGGGTCCTGTTTCAAAGTCAGAACAAGAAAGAAATTGCCCTTTGGCGATCAATACCTTCTAAACCCGGTT
TTGGCCTATCAACTGACTTCCAGACGACTGAATTCCTGGAATGTCTGAAGCAAGAGGCTGGCGCACCAAGC
ATGGAAGAACTTTGTAATAATTACAAGGAATATTTGCGCCCAACTGACTGTTCTGGTTTCGACTGGTCAGTCG
CCTTCTGGATGCTCGAGGACGATATGGAGGTGAGAAATCGCCTAACCTACAACAACACCGAGCTCACCAAG
CGCCTACGGGCAGCATGGTTGAAGTGTATAGGAAATTCTGTCCTATGCCTATCCGATGGCACCTTGCTAGCTC
AACAAGTCCCTGGAGTACAAAAGAGTGGAAGCTACAATACTAGCTCCTCGAATTCAAGAATCCGGGTTATGG
CAGCCTATCATTGTGGCGCCGATTGGGCAATGGCCATGGGGGACGATGCTTTAGAGAGCCCCCACTCCAAC
CTAGAGGAGTATAAAAATTTAGGTTTCAAAGTCGAGGTGAGTCGAGAACTCGAATTCTGTTCACATATTTTCA
GAACTCCGACCCTCGCCATTCCGGTCAACACCAACAAAATGCTTTACAAGTTGATCCATGGGTATAACCCGG
AATGTGGCAACCCAGAAGTTATAGCAAACTATCTGGCTGCGGTATTTTCAGTACTGCATGAACTTCGATATGA
TCCGGAGTTAGTTGCCAGGCTTCACCAGTGGTTGGCTCCGAGTGCCACCACAAAAGAACACTGAGAGAAG
CCTCGATAAGCTAGCCAAACATACACAAGTTGCAAGTGTTGGAAGCTATAGTCTTGTTACCAAAGCCGAACA
CAATAGATTACAAGTTTCTAGCTGGATTTGCATCAGGTTTTCTAACCGCAATCCCAATTTCAGTAGTCGGCATA
TATATAGTCTACCTAAGAATCTCCTCACACGTAAGATCAATTGTTAATGAATACGGTCGTGGGTAGGAGAACAA
TCAATGGAAGAAGACGACCACGCAGGCAAACAAGGCGCATTCAGCGAAATCAGCCAGTGGTTGTGGTCCA
AACCTCTCGGCCAGCACAACGCCGATCTAGACGACGACGAAGAGGTAACAACCGGACAGGAAGAGCTGTT
CCTACCAGAGGAGCTGGTTCGAGCGAGACATTTGTTTTCTCAAAAGACAATCTCGCGGGAAGTTCCAGCGG
AGCAATCACGTTCGGGCCGAGTCTATCAGACTGCCCGGCATTCGCTAATGGAATGCTCAAGGCCTACCATGA
GTATAAAATCTCAATGGTCATTTTGGAGTTCATCTCCGAGGCCTCTTCCCAAAGTTCCGGTTCCATCGCTTACG
AGCTGGACCCACACTGTAAACTCAATTCCCTTTCCTCAACAATCAATAAATTCGGGATCACAAAGCCCGGGA
AAAGGGCGTTTACAGCGTCTTACATCAACGGGGCGGAATGGCACGACGTTGCCGAGGACCAATTCAGGAT
CCTCTACAAAGGCAATGGTTCCTCATCGCCAGCTGGTTCTTTCAGAATCACCATAAAGTGTCAATTCCATAAC
CCCAAATAGGTAGACGAGGAACCTAGCCCTAGTCCAGGGCCAAGCCCCCAACCAACACCAACACCAACCAA
AGAGTACAGATGGATAGTTTACACTGGCGTCTGCAGAACTCTAATAAAAGCTCAGACAACAGACGATTCAAT
TTATCTGTATGATCTGGGTTCCCAACGCCTTCGGTATATTGAAAATGAAAACATGAACTGGACCAACGTGGAC
GCCCGCTGGTATTCAAACAGCAATGTTAAAGCGGTTCCCATGTATGTGTTTCCGGTCCCGGAAGGAGCATGG
TCAGTCGAGATATCAACAGAAGGTTACCAACCTACAGCGAGTACCACTGACCCAAATAAGGGAAAGGTTGA
TGGCATGATTGCCTATTCTGATGATCGATCAGAAGTGTGGAATGTTGGAATAAATCAAAACTGTAACATCACA
AACCTGAAAGCAGATAATTCCTGGAAGTATGGGCACCCTGATATGGAAATAAACAATTGCCACTTCAACCAG
GGACAGGTATTGGAAATGGATGGTACAGTCTCCTTTCACGTTGAGACCACTGGTGCCGATGCATCATTTTTC
CTGGTTGGTCCAGCAGTCCAAAAGTTGTCAAAATACAATTACGCCGTCTCATACGGAGCGTGGACAGACCGT
GATATGGAGTTGGGCTTGATTACAGTGTCTTTGGATGAAAAAGATGAATCTAGAGGTTCCGCCCTTAAAAGA
CCTAGTCGAGAAGGACACTCGAAGGCAGTCTCCACCTGGGAGACTATAAACTTACCGGAGAAGGAAAACTC
CGATGAAACTAAAACCAGTCAAAGACAAGACTTTAACACTCCTCTCACAGCTGGTGGGAGTTCTGATATGCT
GGATATCTATGAAGGAGGCTCACCCTTCCCTGCTGAAGAGGACATTCCCGACTTTGTTGAGGATGACCCATG
GTCTGACATTTCGACTAATAAGTTGCAGGAGGAGGAGGTTATGTCCACAAGGAGTGGTCTTACACCACAGTT
AAAGCCTCCTGGTCTGCCAAAACCCCAACCGGTGAGAACAATTTCAAGTTTCAATCCAACGCCGGATTTGGT
TGAAGCATGGCGACCTGATGTGAACCCTGGTTATTCCAAAGAAGACGTGGCTGCTGCCACCGTAATCGCAG

GGGGTTCTATAAAAGACGGCCGGTCTATGATCAACAAGCGCGACAAAGCTGTGTTAGACGGTCGCAAGAGT
TGGGGTTCTTCCTTAGCTTCTTCCTTGACAGGGGGAACGCTCAAAGCTTCCGCTAAATCTGAGAAACTTGCG
AAACTCACTACTAGTGAGAGAGCGGAATATGAACGGATTAAGCGTCAGCGAGGTGCCACACAAGCTTCAGA
GTTTTTAGAACAACTTTTAGCTGGCAAAGACTCCAACCCAAGGCTCTGAAGAGAGACAGCCTGACTCTTCC
CGGTCCTGATGAACCTGTCCAAATCATCACCGTCAAGCCCGTGACGTTAAACGTGGAACGACTCCGATAGGA
TAGGCACGAGTGTTTTACGCTGGGGTAAATCCCCTACGGCACTTCGGTGT
>SeqID [organism=Tobacco polerovirus virus 1] [moltype=genomic RNA] [isolate=YKMPL] tobacco polerovirus virus 1 isolate YKMPL
CAGGAGGAATTGCAGCTCTAAGCGCAACTCTTTACACTCATTGCAATCGTTATAGCACATGTTTGTATTGACA
CCAACAGGATCAATCGCATTTGACGAAACCAAGAAGCTAACAAAGCTAGTTTTTGTCAATTTAGTGATTGGA
TTTCCTACCCTCCTAGCTCAAGCTCACATTCTTTTTCACTACAATTATGAACAAATTTATAACATTTCTCGTAGT
GTTTTCTATCTACTCCCTCTGCTCAGCAGATGCAAAGGCTGGATTTCATCATCCGGCTTATCTATATCGAGGCA
CTGGCATAGCCAATGTCTCAAATGGGGGTTACTATGCGGCACCCATCCCGCTATACAAATTGGGCACACTTCC
CTCACCATCAAAATTGGTGAGCCCTCCTCCGCTGCAGCTTACAGACGCGAGCTATCAAGAATTGATTCAAGC
TCTTACGTCCAAAATGCGCGCGGACTCTACAACTCTTGGGGACAAGACATGGCAACATTTGTCAAAAATGCT
GTGTGTTTCCTTGAAATCCGCAAAAGATGCTACCCGAAGCGCGGTCTCAACGCTACTCTGGACTATAATTTCA
ATTTGGTTCGGAGTTTATTGGACGCTTGCCAAGATACTGGCCATCTTCTTGTGGACGTTCAGTATACAGTCCT
TTTGCATAATCTTGCTGTGTTTTACAACCAGCTTGATTTTCAAGGCTGTGAAATTCTGTTTCGAGAGCTTACC
GGTTTACCTGTTTACGTACCCGGCGAAGCTTATTTGGAGGGCAGCTTTCTCCAAAAAGAATTACAAGGATGA
GAAAGCCGTAGAGGGTTTTAGAAGTTTCGTGGTCCCTCAAAAACCACCAAAGTCTGCCGTGATTGAATTACA
ACATGAAAACGGCAGCCACCTGGGTTATGCTAATTGCATAAGATTATATAGTGGAGAGAACGCGCTTGTAAC
AGCAGAACACTGCTTGCAGGGTGCGTACGCAGCATCGATGAAAACTGGCAATAGGATTCCGATGTCTAGCT
TCATTCCCATTTACAAAAGCAACCCACGTGACATCGCCATCATGGCAGGACCCCCAAATTGGGAAGGGCTTC
TTGCTGTCAAAGGCGCCAGTTTCCAAACTGCTGACAAAATCGGCAGAGGACCTGCTTCTTTCTTCACGCTAG
AGAAAGGAGAGTGGATGTGCAACAATGCCCAAATAGATGGATCACATGACAAATTTGTGACTGTCTTGTGTA
ACACTGAAGCAGGGTGCTCAGGGACAGGGTTTTGGTCTTCCAAAACTTTGTTGGGCGTGCTCAAAGGATAC
CCACTAGAGGAAGGGTGCAATTACAACGTTATTTCTGTAATACCTTCCATTCCAGGTCTGACCTCCCCTAACTA
CGTGTTTGAATCGACTACCATAAGAGGTAGAGTCTTCAGTTCAGAAGCAATCGAAGAGATGGAGAGGGAG
GCTAGGGAGGCAGTGAGAAAGTTGCTGTCCTTCAAGTCTCAAACTGGAAAGAACTGGGCCGATTACTCCGA
TGATGAGGAGTATGGTGATGAAAAGAAGGCAGCTGAAGCGCCAAAGAAGGAAGCTCAAGCGCCCAAGGA
GGAAACTAAAGAGGTTCCCGCAGAGAAAACTGCGCAAACAAACCCACAGGCTCCTTTAAATGGGGTCGCG
GCACCGCCCGCCATAACAACCGGAACCTCCGCCACCCCAAGCGGCGCTACCAGCGCACCACCGATGGACAA
GATGGTCGAGCAGATCATCACAGCTATGGTGGGGAGGATAAACCTCTCGGAAATCGAGAGGAAAATAGTGG
AAAGAGTTTCAGAGAAAGCTCTCAAGAAGCCAACTCAAAGCAAGCGAGGAAGGCGTGGAGGGAAGAACA
AGCCAGACAATTCATCTCCTACTTCCGAGCCATCTACACGTGGGGCGCCCAAGAGGGTGCAGCCCCCCCAG
GCTTCAAAAGATGCGGTAGGACCCCGCGTTACTACCACCCCCGTACCAGAGGTGAAACCCAATGGGGGCGC
AAACTCTGCCAAGTTCATCCCGAGCTGGCGAATAAAACAGCAGGCTTCGGCTGGCCAAAGACCGGAGCAC
AAGCAGAACTCCAAAGCTTGAATCTGCAGGCTGCTAGGTGGCTCGAACGTGCCGAGTCGTCCACTATACCTA
GTGCAGAAGCGAGAGAGCACGTGATTCAAAAGACTGTGAGAGCGTACCAAAACTGTACAACTCAAGCTCC
ACAGTGCTCCCTTAAATCCAAACTGGACTGGACTGGCTTCCAAAATGATATAAAAGAAGCAGTCCGGTCTCT
TGAATTAGATGCGGGTGTAGGCATCCCTTACATAGCATATGGGCTCCCCACTCACCGAGGATGGGTTGAAGA
TCCCAAGCTCCTGCCGATAATTGCTCAGTTGACCTTCGACCGACTACAGAAGATGTCGGAGGCCAACTTCGA
GTCCATGACTCCGGAGGAGCTCGTTCAAGAGGGGCTTTGCGATCCTATAAGATTATTTGTCAAGGGTGAGCC
CCATAAGCAGAGCAAACTGGATGAAGGCCGCTACCGCCTCATTATGTCAGTTTCTTTAATAGATCAACTGGTA

GCCCGGGTCCTGTTTCAAAGTCAGAACAAGAAAGAAATTGCCCTTTGGCGATCAATACCTTCTAAACCCGGT
TTTGGCCTATCAACTGACTTCCAGACGACTGAATTCCTGGAATGTCTGAAGCAAGAGGCTGGCGCACCAAG
CATGGAAGAACTTTGTAATAATTACAAGGAATATTTGCGCCCAACTGACTGTTCTGGTTTCGACTGGTCAGTC
GCCTTCTGGATGCTCGAGGACGATATGGAGGTGAGAAATCGCCTGACCTACAACAACACCGAGCTCACCAA
GCGCCTACGGGCAGCGTGGTTGAAGTGTATAGGAAATTCTGTCCTATGCCTATCCGATGGCACCTTGCTAGCT
CAACAAGTCCCTGGAGTACAAAAGAGTGGAAGCTACAATACTAGCTCCTCGAATTCAAGAATCCGGGTTATG
GCAGCCTATCATTGTGGCGCCGATTGGGCAATGGCCATGGGGGATGATGCTTTAGAGAGCCCCCACTCCAA
CCTAGAGGAGTATAAAAATTTAGGTTTCAAAGTCGAGGTGAGTCGAGAACTCGAATTCTGTTCACATATTTTC
AGAACTCCGACCCTCGCCATTCCGGTCAACACCAACAAAATGCTTTACAAGTTGATCCATGGGTATAACCCG
GAATGTGGCAACCCAGAAGTTATAGCAAACTATCTGGCTGCGGTATTTTCAGTACTGCATGAACTTCGATATG
ATCCGGAGTTAGTTGCCAGGCTTCACCAGTGGTTGGCTCCGAGTGCCACCACAAAAGAACACTGAGAGAA
GCCTCGATAAGCTAGCCAAACATACACAAGTTGCAAGTGTTGGAAGCTATAGTCTTGTTACCAAAGCCGAAC
ACAATAGATTACAAGTTTCTAGCTGGATTTGCATCAGGTTTTCTAACCGCAATCCCAATTTCAGTAGTCGGCAT
ATATATAGTCTACCTAAGAATCTCCTCACACGTAAGATCAATTGTTAATGAATACGGTCGTGGGTAGGAGAACA
ATCAATGGAAGAAGACGACCACGCAGGCAAACAAGGCGCATTCAGCGAAATCAGCCAGTGGTTGTGGTCC
AAACCTCTCGGCCAGCACAACGCCGATCTAGACGACGACGAAGAGGTAACAACCGGACAGGAAGAGCTGT
TCCTACCAGAGGAGCTGGTTCGAGCGAGACATTTGTTTTCTCAAAAGACAATCTCGCGGGAAGTTCCAGCG
GAGCAATCACGTTCGGGCCGAGTCTATCAGACTGCCCGGCATTCGCTAATGGAATGCTCAAGGCCTACCATG
AGTATAAAATCTCAATGGTCATTTTGGAGTTCATCTCCGAGGCCTCTTCCCAAAGTTCCGGTTCCATCGCTTAC
GAGCTGGACCCACACTGTAAACTCAATTCCCTTTCCTCAACAATCAATAAATTCGGGATCACAAAGCCCGGG
AAAAGGGCGTTTACAGCGTCTTACATCAACGGGGCGGAATGGCACGACGTTGCCGAGGACCAATTCAGGA
TCCTCTACAAAGGCAATGGTTCCTCATCGCCAGCTGGTTCTTTCAGAATCACCATAAAGTGTCAATTCCATAAC
CCCAAATAGGTAGACGAGGAACCTAGCCCTAGTCCAGGGCCAAGCCCCCAACCAACACCAACACCAACCAA
AGAGTACAGATGGATAGTTTACACTGGCGTCTGCAGAACTCTAATAAAAGCTCAGACAACAGACGATTCAAT
TTATCTGTATGATCTGGGTTCCCAACGCCTTCGGTATATTGAAAATGAAAACATGAACTGGACCAACGTGGAC
GCCCGCTGGTATTCAAACAGCAATGTTAAAGCGGTTCCCATGTATGTGTTTCCGGTCCCGGAAGGAGCATGG
TCAGTCGAGATATCAACAGAAGGTTACCAACCTACAGCGAGTACCACTGACCCAAATAAGGGAAAGGTTGA
TGGCATGATTGCCTATTCTGATGATCGATCAGAAGTGTGGAATGTTGGAATAAATCAAAACTGTAACATCACA
AACCTGAAAGCAGATAATTCCTGGAAGTATGGGCACCCTGATATGGAAATAAACAATTGCCACTTCAACCAG
GGACAGGTATTGGAAATGGATGGTACAGTCTCCTTTCACGTTGAGACCACTGGTGCCGATGCATCATTTTTC
CTGGTTGGTCCAGCAGTCCAAAAGTTGTCAAAATACAATTACGCCGTCTCATACGGAGCGTGGACAGACCGT
GATATGGAGTTGGGCTTGATTACAGTTTCTTTGGATGAAAAAGATGAATCTAGAGGTTCCGCCCTTAAAAGA
CCTAGTCGAGAAGGACACTCGAAGGCAGTCTCCACCTGGGAGACTATAAACTTACCGGAGAAGGAAAACTC
CGATGAAACTAAAACCAGTCAAAGACAAGACTTTAACACTCCTCTCACAGCTGGTGGGAGTTCTGATATGCT
GGATATCTATGAAGGAGGCTCACCCTTCCCTGCTGAAGAGGACATTCCCGACTTTGTTGAGGATGACCCATG
GTCTGACATTTCGACTAATAAGTTGCAGGAGGAGGAGGTTATGTCCACAAGGAGTGGTCTTACACCACAGTT
AAAGCCTCCTGGTCTGCCAAAACCCCAACCGGTGAGAACAATTTCAAGTTTCAATCCAACGCCGGATTTGGT
TGAAGCATGGCGACCTGATGTGAACCCTGGTTATTCCAAAGAAGACGTGGCTGCTGCCACCGTAATCGCAG
GGGGTTCTATAAAAGACGGCCGGTCTATGATCAACAAGCGCGACAAAGCTGTGTTAGACGGTCGCAAGAGT
TGGGGTTCTTCCTTAGCTTCTTCCTTGACAGGGGGAACGCTCAAAGCTTCCGCTAAATCTGAGAAACTTGCG
AAACTCACTACTAGTGAGAGAGCGGAATATGAACAGATTAAGCGTCAGCGAGGTGCCACACAAGCTTCAGA
GTTTTTAGAACAACTTTTAGCTGGCAAAGACTCCAACCCAAGGCTCTGAAGAGAGACAGCCTGACTCTTCC
CGGTCCTGATGAACCTGTCCAAATCATCACCGTCAAGCCCGTGACGTTAAACGTGGAACGACTCCGATAGGA
TAGGCACGAGTGTTTTACGCTGGGGTAAATCCCCTACGGCACTTCGGTGT

>SeqID [organism=Tobacco polerovirus virus 2] [moltype=genomic RNA] [isolate=YBSh] tobacco polerovirus virus2 4 isolate YBSh

GGGAGAACACTCATTCTTTCGCTCCTCCGCATTTCATGCAGTTTGAGTTGATTAACGAAAGATATCTCCGAAT
CTCTAGTACGCGCACTCTCTCATACCGAGACCGTTTGCTTAATATTGCAATTTTCTTGCAAAACTTTCTGGTGA
GATTCAACAACAGTTATGAACATAATGAAGCTGTTCGTTCTGTTTGTTTTCTTCTTCCTTTCCTCCTCGGCAAC
GACGATCCCTTCCTGGCAAGGAATACCCCGCACAGGAACGGCAGACGGGAATCTAACCGTATCGCCCGGCT
CTCGCTTTACAGTGGAGTCGGAATCATTGAGCGAAAAACTTCTGCCGCCGCAGCCCATATTTGCCTTGAAAC
AACGGGCAGCCTCCGAGAATCGAGAGATCGAGTTCAACGAAGTGCTGCATCTTCTATGGCACGTTATATTGC
GAGACTGCAGACTCCTCTTTTCCACGGCTCAGCAGATTTTCAAAAATTTCTGCGAGTCTGGACTCACCACTG
CGAAAGCATGGTTGATAGGAGCTTTCGGCGCTTGCCTTTGGGCCCTAATCTCAATATGGAGCTCGTGCATCT
GGGTGGTGATATCATGGATTTACTTTTTGGTCACAACCTACACGATGTACGTCGCCTGCATCGCATTGCTTTAC
GCTTTAACCGCATTTATGGTGAAAGCGTTGCTTTGGACGTTCTCAGGCTGGCCCACTTCCCTCGCACTATTCA
TATACAAAACTGGGAAGAATACTTACACGGCTCTCAGTTACAAAAAGAGCTACGTGGAGGAGAAACAAGTT
AAGGGATTTATTTCTTTGAAAATCCCCCAACAACCCCCAAAATCTTGCATCCTTGAGGTTCAATATGAAGATA
ACTCTCATGCTGGATATGCAAGTTGCGTTCGGCTATTTGATGGCACACTTGGTCTTATGACTGCACGCCACGT
AGTTGACGCTGGTTCCAAGGTAGCATCTGTTAAGAACACCAATAAGATCCCCCTCAGTCAATTCACCCCCCTC
ATAACATCAAATAAGGGTGACTTCATTTTGCTGAGTGGACCTCCTAACTGGGAGAGTCTACTAGCCTGTAAG
GGAGTAGCCTTTGTCCCCGCATCTCAACTGTCAAAATCTAAGATGCGGTTTTACTTCATGGAGAAAGGTGAAT
GGATGGCTGACCACGGCGAGGTCGTAGGACCCCGTGATGCTCACATGGCCGCCACTCTCTGCAATTCTGAG
GCTGGATATTCAGGAACCCCCATCTTTAATGGGAAAATGATAATAGGGGTCCACATCGGGGCTGATAATGATT
TCAACCAGAATCTCATGGCTACAATCCCCCCGGTGTCAGGTTTGACAACCCCCCAGTATGTCTTTGAAACAAC
AGCCCCTCAAGGTAGGGTATTTAATGATGAAGACATAGAGGTAATGCTGAAAAGCATAACAGTCGGAATCCC
TAAGCTTCAAGATTTTAAAAGCATAACAGGGAAAAACTGGGCCGACTATGAAGACGACGATTTCTCCGTCGA
GGTCAAACCCCCCCCCTCTAAAGCTCAATCAGAAGAAGAGGCGGGAAACGAGAAAGGCAGAGCTGTCTGC
CAAACCAACAGCAAAACAGGAGACAAGGTGCCCGAAGCAAGCACCCCACCCGTCAAGCCCACCCCCTTCA
AGCCAACCGACTCTCTCGCAGAAGAAGGAAAATGCTTGGAACAGCTCTTAACGAAGCTGGTCGAGAGGAT
CGACCTTTCCAGCATAGAGAAGAAGGTGGTGGAAGTTCTAGCCGACAAGGCCATGAAGAAACCTCAGAGA
TCCCAGCGGCGAAGAAGACCGCAGAAGGCTTCGAACGATACTTTGCAAGCCAATACAAATGGGAGGTATCA
ACCTCCACACAAGAGATCTCAGGGTTCGAAAGGTGTGGAACCCTCCCCCGCTACTACCAACCAAAACAAATC
AAAAGTAGTGAGTGGGGCTCCCAGCTCATCAAAGAACACCCAGAGCTGGGTGAAAAAGTCTCCGGCTTCG
GCTGGCCGTCAGTAGGCCCCCAAGCTGAGGTGACCTCTCTCACTTTACAAGCAGAGAGGTGGCTGCAACGC
GCGCAGTCAGCTAAAATCCCGTCAACTGAGGACAGGGAGCGCGTGATTCAAAAGACAGTGGGAGCCTACT
CAAATGTTAAAACTACAGGCCCCATTGCTACCCGAGGAAATAAGCTCGAATGGCGGCAATTCATGGAAGATT
TTAAGACTGCCATTTTCTCATTGGAGCTTGATGCAGGTATAGGCGTGCCATACATTGCCTATGGAAGGCCCAC
TCACAAAGGATGGGTGGAAGACCCGAAACTCCTGCCAGTGCTTGCCCACCTCACTTTCAACCGACTACAGA
AGATGTTGGAAGTTGAGTATGAAGATTTAAGTGCTGAACAGCTTGTGCAGGCTGGTTTATGTGATCCCATAA
GGGTCTTTGTTAAAAGAGAACCGCACAAGCAAGCTAAACTCGATGAAGGCCGCTACCGCCTCATCATGAGT
GTTTCCCTAGTAGATCAACTGGTAGCCCGGGTTCTGTTCCAAAATCAGAACAAGCGAGAAATCGCTCTTTGG
AGGGCAAACCCCTCAAAACCCGGTTTTGGCTTGTCTACGGATGAGCAAGTGCTGGAGTTTGTACAGGCTCT
GGCCGCACAAGTGGAAGTAGATCCCTCTGAATTAGTAACAAACTGGGGTGAGTACCTTGTGCCGACTGATT
GCTCTGGTTTTGACTGGAGCGTTGCGGAGTGGATGCTCTATGATGATATGATCGTCCGCAACAAACTTACATC
GGACCTGAATGAAACTACTGAAAAGCTGCGCTTTGCGTGGTTAAAGTGCATAAGCAACAGTGTCCTTTGTTT
GAGTGATGGCACCCTCCTTGCCCAAAGAATCCCGGGAGTTCAAAAGTCTGGAAGCTACAATACAAGCAGCT
CCAACTCTCGGATTCGAGTCATGGCCGCATACCATTGTGGAGCCGACTGGGCGATTGCTATGGGGGATGATG

CCCTTGAATCAGTCAACACCAACCTAGAGGTGTATAAAAGTCTAGGTTTTAAAGTCGAGGTTTCAGGACAAC
TGGAATTCTGCTCTCACATTTTTAGAGCGCCTGACCTCGCCCTCCCAGTGAATGAGCGTAAAATGCTGTACAA
GCTCATCTTCGGTTACAATCCTGGGAGCGGGAGTCTGGAGGTGATCTCCAACTATATTGCCGCCTGTGCATCC
GTGTTAAACGAGTTGCGGCATGACCCAGATTCAGTTGCTATTCTCTACCAGTGGCTAGTTAATCCAGTGCTGC
CACAAAACGATTAAAAGGAGAAAGCAAGATAACCAGCCAAGCATACATCAGTTGCAAGCATTGGAGGTTTC
AGTCTGATTACCAAAGCCTGACAAGATAGATTATAAATTTCTTAGTGGATTTGCTTTAGGATTCTTATCCTCAAT
CCCAATTTCGATAGCAGGCTTGTACTTTATCTACCTAAAGATTTCAGCCCACGTACGCGCGATCGTTAATGAAT
ACGGGCGGAGCTAGGAGAAATAACAATGGAAATGGTGGATCACGAAGCACCCGCCGTCGCAGACGCCCAA
GACAGGTTCGCCCTGTCGTTGTGGTCGCACCCTCTGGGACAGCACGGCGTGGAGGTCGAAGACGACGAAG
TGGAGGCCGGAACCGAAGAAGCCGAGATGGAATTGGAGGAAGGTCAAGCAACAGCGAGACTTTCATCTTC
AACAAGGACTCAATCAAGGATAGTTCCTCAGGCTCAATCACTTTCGGGCCGTCTCTATCAGAGAGCGTCGCG
CTTTCAGGTGGAGTTCTCAAAGCCTACCATGAATATAAGATCACAATGGTCAACATACGCTTCATCAGTGAAT
CCTCTTCCACAGCGGAGGGCTCCATCGCTTACGAGCTGGACCCCCACTGCAAGCTTTCTAGTCTCCAATCAA
CCCTCCGTAAATTCCCCGTCACCAAAGGCGGGCAAGCAACGTTCAGGGCTGCGCAAATTAATGGGGTAGAG
TGGCATGATACAACCGAAGATCAATTTAGGCTGCTCTATAAAGGCAACGGAACAAAGAACGTTGCCGCCGG
GTTTTTCCAAATCCGGTACACTGTACAATTGCACAACCCCAAATAGGTAGAATCCGAACCAGGACCGAGCCC
CGGGCCTCAGCCAGAGCCCACACCCACTCCCTCTCCTCAAAAGCACGAGCGCTTCATCGCCTATGTCGGTAT
CCCAATGCTGACTATTCAAGCTAAGGAGAACGACGACCAGATCTTGCTAAGATCCATGGGACCTCAAAGAAT
GAAATACATCGAGGATGAGAATCAGAACTATACGAATATAGATTCGCAGTTTTATTCTCAGAGCAGCATGAGT
GCTGTCCCCATGTACTATTTTAATGTGCCAAAGGGCCAATGGTCAGTCGATATCAGCTGTGAAGGTTACCAGC
CTACAAGCAGCACCACAGATCCCAATCGTGGTAGGAGTGATGGTTTGATTGCATACTCCAATAGTGACTCAG
ATTACTGGAATGTGGGAGAAGCGGATGGTGTTAAAATTTCCAATTTAAGGAATGATAACACGTACCGCCAAG
GACATCCAGATCTTGAGATCAATTCCTGTCATTTTAGAGACGGGCAACTGTTAGAACGTGACGCAACTATAA
GTTTCCACGTAGAAGCTCCAGAAGATGGACGCTTCTTTCTGATAGGGCCTGCAATCCAAAAGACCGCCAAGT
ACAATTATACTATTTCTTATGGCGAGTGGACTGACAGAGACATGGAGTTGGGCTTAATAACAGTTGTGCTAGA
TGAGCACCTGGAGAGTTCTGGTTCGGCAAGAAGAATGAGGGGGGCCGTGCGACACGCGCGACATCATCAG
CCCCACCCTCCTCCGGAGGAGAAACCGGAAAACAAACCCTGGAAAGAAAACCAGTCTGAAACTCGGGAAA
GACAACCCGTTAAAACTCCATCTCCGGATGTCTCTGAGGCTGGCTCAGATCAAGATGACGAGACCAAACCTT
GGGATCTCATCGAGAAGGTTAGCAAGGGAGAGCCTTTGGATTCGTTTCAACAGCTTGAGTATGATAAGTACC
GCTTTAGAGAATCGAATATCATTGAGCTAAATGAAGAAGATAATTCAAGTCAACACCCTTTGCTACCACCTCC
CATCCCTCACAAAACGCTGCCCCCAGGTCGCACTGAAACAGACTTCAAACCTGAAGCCAATGCACTAGAAG
CCTGGGACAAACAGCACTTCGACCCAGGATACTCCAGGGAAGAAGTAGCGGCAGCCACTATAGTCGCCGG
AGGCTCCATAGCCGACGGTCGCAGCGCTCTGCAGGAAAGAGAAGCTAAAATACAGCGTGCTCGGACCACTT
GGTCTGACGAGCAGAAGCCATTTAACCCTGCTATAGCGAAGCTGAGAGGAGTTAGAGCTGAAGAAAGGAA
AGTTCTGACTGGTGGATCTTTAAGAGGCGGGTCTGACACTGCTTCCAGTTTGGGGGGCGGATCCCTGTCTG
GTGGGACGTTGAAGCCAAAGAAAACTTTAGAGCAAAGCGTGGTGGAAACTATGTCGACTGCTCAAAGGTT
GGAATACGAAAGGTTGAAAAGTGTTAGCGATTCTGAAGCTAACAAATATCTGTGGAACTTCAAACCCCCACC
CACACCACCGAAAGGACCAGATAAGTTTGGTAGGAGGTAGTCTAAGCTCTATGACTTTAAAATAACGAGCAC
ACTGAACTCCAAGCCTGTTAGAGTATAACCCAGGTCGTCATTAGCTGTAGACGTAAAACCAGCTGCACCGCG
CGGTTGGCCGTCGAGCGCGAGATAATAAACAATGGCAACGAAAACTGAAGTCTGAGTAAGTTAGTAGGCAC
TATAAAGTATAAATTCAGG

>SeqID [organism=Tobacco polerovirus virus 2] [moltype=genomic RNA] [isolate=YKMPL] tobacco polerovirus virus 2 isolate YKMPL

GGGAGAACACTCATTCTTTCGCTCCTCCGCATTTCATGCAGTTTGAGTTGATTAACGAAAGATATCTCCGAAT

CTCTAGTACGCGCACTCTCTCATACCGAGACCGTTTGCTTAATATTGCAATTTTCTTGCAAAACTTTCTGGTGA
GATTCAACAACAGTTATGAACATAATGAAGCTGTTCGTTCTGTTTGTTTTCTTCTTCCTTTCCTCCTCGGCAAC
GACGATCCCTTCCTGGCAAGGAATACCCCGCACAGGAACGGCAGACGGGAATCTAACCGTATCGCCCGGCT
CTCGCTTTACAGTGGAGTCGGAATCATTGAGCGAAAAACTTCTGCCGCCGCAGCCCATATTTGCCTTGAAAC
AACGGGCAGCCTCCGAGAATCGAGAGATCGAGTTCAACGAAGTGCTGCATCTTCTATGGCACGTTATATTGC
GAGACTGCAGACTCCTCTTTTCCACGGCTCAGCAGATTTTCAAAAATTTCTGCGAGTCTGGACTCACCACTG
CGAAAGCATGGTTGATAGGAGCTTTCGGCGCTTGCCTTTGGGCCCTAATCTCAATATGGAGCTCGTGCATCT
GGGTGGTGATATCATGGATTTACTTTTTGGTCACAACCTACACGATGTACGTCGCCTGCATCGCATTGCTTTAC
GCTTTAACCGCATTTATGGTGAAAGCGTTGCTTTGGACGTTCTCAGGCTGGCCCACTTCCCTCGCACTATTCA
TATACAAAACTGGGAAGAATACTTACACGGCTCTCAGTTACAAAAAGAGCTACGTGGAGGAGAAACAAGTT
AAGGGATTTATTTCTTTGAAAATCCCCCAACAACCCCCAAAATCTTGCATCCTTGAGGTTCAATATGAAGATA
ACTCTCATGCTGGATATGCAAGTTGCGTTCGGCTATTTGATGGCACACTTGGTCTTATGACTGCACGCCACGT
AGTTGACGCTGGTTCCAAGGTAGCATCTGTTAAGAACACCAACAAGATCCCCCTCAGTCAATTCACCCCCCT
CATAACATCAAATAAGGGTGACTTCATTTTGCTGAGTGGACCTCCTAACTGGGAGAGTCTACTAGCCTGTAAG
GGAGTAGCCTTTGTCCCCGCATCTCAACTGTCAAAATCTAAGATGCGGTTTTACTTCATGGAGAAAGGTGAAT
GGATGGCTGACCACGGCGAGGTCGTAGGACCCCGTGATGCTCACATGGCCGCCACTCTCTGCAATTCTGAG
GCTGGATATTCAGGAACCCCCATCTTTAATGGGAAAATGATAATAGGGGTCCACATCGGGGCTGATAATGATT
TCAACCAGAATCTCATGGCTACAATCCCCCCGGTGTCAGGTTTGACAACCCCCCAGTATGTCTTTGAAACAAC
AGCCCCTCAAGGTAGGGTATTTAATGATGAAGACATAGAGGTAATGCTGAAAAGCATAACAGTCGGAATCCC
TAAGCTTCAAGATTTTAAAAGCATAACAGGGAAAAACTGGGCCGACTATGAAGACGACGATTTCTCCGTCGA
GGTCAAACCTCCCCCCTCTAAAGCTCAATCAGAAGAAGAGGCGGGAAACGAGAAAGGCAGAGCTGTCTGC
CAAACCAACAGCAAAACAGGAGACAAGGTGCCCGAAGCAAGCACCCCACCCGTCAAGCCCACCCCCTCCA
AGCCAACCGACTCTCTCGCAGAAGAAGGAAAATGCTTGGAACAGCTCTTAACGAAGCTGGTCGAGAGGAT
CGACCTTTCCAGCATAGAGAAGAAGGTGGTGGAAGTTCTAGCCGACAAGGCCATGAAGAAACCTCAGAGA
TCCCAGCGGCGAAGAAGACCGCAGAAGGCTTCGAACGATACTTTGCAAGCCAATACAAATGGGAGGTATCA
ACCTCCACACAAGAGATCTCAGGGTTCGAAAGGTGTGGAACCCTCCCCCGCTACTACCAACCAAAACAAATC
AAAAGTAGTGAGTGGGGCTCCCAGCTCATCAAAGAACACCCAGAGCTGGGTGAAAAAGTCTCCGGCTTCG
GCTGGCCGTCAGTAGGGCCTTCAGCCGAGGTGACCTCTCTCACTTTGCAAACAGAGAGGTGGCTGCAACG
CGCGCAGTCAGCTAAAATCCCGTCAACTGAGGACAGGGAGCGCGTGATTCAAAAGACAGTGGGAGCCTAC
TCAAATGTTAAAACTACAGGCCCCATTGCTACCCGAGGAAATAAGCTCGAATGGCGGCAATTCATGGAAGAT
TTTAAGACTGCCATTTTCTCATTGGAGCTTGATGCAGGTATAGGCGTGCCATACATTGCCTATGGAAGGCCCA
CTCACAAAGGATGGGTGGAAGACCCGAAACTCCTGCCAGTGCTTGCCCACCTCACTTTCAACCGACTACAG
AAGATGTTGGAAGTTGAGTATGAAGATTTAAGTGCTGAACAGCTTGTGCAGGCTGGTTTATGTGATCCCATA
AGGGTCTTTGTTAAAAGAGAACCGCACAAGCAAGCTAAACTCGATGAAGGCCGCTACCGCCTCATCATGAG
TGTTTCCCTCGTTGATCAATTGGTAGCCCGGGTTTTATTCCAGAGTCAGAACAAGCGAGAAATCGCACTTTG
GAGGGCCAATCCCTCAAAACCTGGTTTTGGCTTGTCTACGGATGAGCAAGTGCTGGAGTTTGTACAGGCTC
TGGCCGCACAAGTGGAAGTAGATCCCTCTGAATTAGTAACAAACTGGGGTGAGTACCTTGTGCCGACTGATT
GCTCTGGTTTTGACTGGAGCGTTGCGGAGTGGATGCTCTATGATGATATGATCGTCCGCAACAAACTTACATC
GGACCTGAATGAAACTACTGAAAAGCTGCGCTTTGCGTGGTTAAAGTGCATAAGCAACAGTGTCCTTTGTTT
GAGTGATGGCACCCTCCTTGCCCAAAGAATCCCGGGAGTTCAAAAGTCTGGAAGCTACAATACAAGCAGCT
CCAACTCTCGGATTCGAGTCATGGCCGCATACCATTGTGGAGCCGACTGGGCGATTGCTATGGGGGATGATG
CCCTTGAATCAGTCAACACCAACCTAGAGGTGTATAAAAGTCTAGGTTTTAAAGTCGAGGTTTCAGGACAAC
TGGAATTCTGCTCTCACATTTTTAGAGCGCCTGACCTCGCCATCCCCGTGAATGAGCGTAAAATGCTGTACAA
GCTCATCTTTGGCTACAATCCGGGGAGTGGGAGTCTGGAAGTGATTTCTAATTACATTGCCGCCTGTGCATCC

GTGTTAAACGAGTTGCGGCATGACCCAGATTCAGTTGCTATTCTCTACCAGTGGCTAGTTAATCCAGTGCTGC
CACAAAACGATTAAAAGGAGAAAGCAAGATAACCAGCCAAGCATACATCAGTTGCAAGCATTGGAGGTTTC
AGTCTGATTACCAAAGCCTGACAAGATAGATTATAAATTTCTTAGTGGATTTGCTTTAGGATTCTTATCCTCAAT
CCCAATTTCGATAGCAGGCTTGTACTTTATCTACCTAAAGATTTCAGCCCACGTACGCGCGATCGTTAATGAAT
ACGGGCGGAGCTAGGAGAAATAACAATGGAAATGGTGGATCACGAAGCACCCGCCGTCGCAGACGCCCAA
GACAGGTTCGCCCTGTCGTTGTGGTCGCACCCTCTGGGACAGCACGGCGTGGAGGTCGAAGACGACGAAG
TGGAGGCCGGAACCGAAGAAGCCGAGATGGAATTGGAGGAAGGTCGAGCAACAGCGAGACTTTCATCTT
CAACAAGGACTCAATCAAGGATGGTTCCTCAGGAGCTATCACCTTCGGGCCGTCTCTATCAGAGAGCGTCGC
GCTTTCAGGTGGAGTTCTCAAAGCCTACCATGAGTATAAGATCACAATGGTCAACATACGCTTCATCAGTGAA
TCCTCTTCCACAGCGGAGGGCTCCATCGCTTACGAGTTGGACCCCCACTGCAAGCTTACTAGCCTCCAATCC
ACCCTGCGTAAGTTCCCCGTCACCAAAGGCGGGCAAGCTACGTTCCGGGCTGCGCAGATTAATGGGGTAGA
GTGGCATGATACAGCTGAAGATCAATTTAGGCTGCTCTATAAAGGCAATGGAACAAAGAACGTTGCCGCCG
GGTTTTTCCAAATCCGGTACACTGTACAATTGCACAACCCCAAATAGGTAGAATCCGAACCAGGACCGAGCC
CCGGGCCTCAGCCAGAGCCCACACCCACTCCCTCTCCTCAAAAGCACGAGCGCTTCATCGCCTATGTCGGTA
TCCCAATGCTGACTATTCAAGCTAAGGAGAACGACGACCAGATCTTGCTAAGATCCATGGGACCTCAAAGAA
TGAAATACATCGAGGATGAGAATCAGAACTATACGAATATAGATTCGCAGTTTTATTCTCAGAGCAGCATGAG
TGCTGTCCCCATGTACTATTTTAATGTGCCAAAGGGCCAATGGTCAGTCGATATCAGCTGTGAAGGTTACCAG
CCTACAAGCAGCACCACAGATCCCAATCGTGGTAGGAGTGATGGTTTGATTGCATACTCCAATAGTGACTCA
GATTACTGGAATGTGGGAGAAGCGGATGGTGTTAGAATTTCCAATTTAAAGAATGATAACACGTACCGCCAA
GGACACCCAGATCTTGAGATTAACACCTGTCATTTTAGAGACGGGCAACTGTTAGAACGTGACGCAACTATA
AGTTTCCACGTAGAAGCTCCAGAAGATGGACGCTTCTTTCTGATAGGGCCTGCAATCCAAAAGACCGCCAA
GTACAATTATACTATTTCTTATGGCGAGTGGACTGACAGAGACATGGAGTTGGGCTTAATAACAGTTGTGCTA
GATGAGCACCTGGAGAGTTCTGGTTCGGCAAGAAGAATGAGGGGGGCCGTGCGACACGCGCGACATCATC
AGCCCCACCCTCCTCCGGAGGAGAAACCGGAAAACAAACCCTGGAAAGAAAACCAGTCTGAAACTCGGGA
AAGACAACCCGTTAAAACTCCATCTCCGGATGTCTCTGAGGCTGGCTCAGATCAAGATGACGAGACCAAACC
TTGGGATCTCATCGAGAAGGTTAGCAAGGGAGAGCCTTTGGATTCGTTTCAACAGCTTGAGTATGATAAGTA
CCGCTTTAGAGAATCGAATATCATTGAGCTAAATGAAGAAGATAATTCAAGTCAACACCCTTTGCTACCACCT
CCCATCCCTCACAAAACGCTGCCCCCAGGTCGCACTGAAACAGACTTCAAACCTGAAGCCAATGCACTAGA
AGCCTGGGACAAACAGCACTTCGACCCAGGATACTCCAGGGAAGAAGTAGCGGCAGCCACTATAGTCGCC
GGAGGCTCCATAGCCGACGGTCGCAGCGCTCTGCAGGAAAGAGAAGCTAAAATACAGCGTGCTCGGACCA
CTTGGTCTGACGAGCAGAAGCCATTTAACCCTGCTATAGCGAAGTTGAGAGGAGTTAGAGCTGAAGAAAGG
AAAGTTCTGACTGGTGGATCTTTAAGAGGCGGGTCTGACACTGCTTCCAGTTTGGGAGGCGGATCCCTGTC
TGGTGGGACGTTGAAGCCAAAGAAAACTTTAGAGCAAAGCGTGGTGGAAACTATGTCGACTGCTCAAAGG
TTGGAATACGAAAGGTTGAAAAGTGTTAGCGATTCTGAAGCTAACAAATATCTGTGGAACTTCAAACCCCCA
CCCACACCACCGAAAGGACCAGATAAGTTTGGTAGGAGGTAGTCTAAGCTCTATGACTTTAAAATAACGAGC
ATACTGAACTCCAAGCCTGTTAGAGTATAACCCAGGTCGTCATTAGCTGTAGACGTAAAACCAGCTGCACCG
CGCGGTTGGCCGTCGAGCGCGAGATAATAAACAATGGCAACGAAAACTGAAGTCTGAGTAAGTTAGTAGGC
ACTATAAAGTATAAATTCAGGAGGGC
